# The Golgi complex governs natural killer cell lytic granule positioning to promote directionality in cytotoxicity

**DOI:** 10.1016/j.celrep.2024.115156

**Published:** 2025-01-14

**Authors:** Luis A. Pedroza, Frederique van den Haak, Alexander Frumovitz, Evelyn Hernandez, Everardo Hegewisch-Solloa, Tabitha K. Orange, Keri B. Sheehan, Susan Prockop, Aaron Bodansky, Ivan K. Chinn, James R. Lupski, Jennifer E. Posey, Emily M. Mace, Yu Li, Jordan S. Orange

**Affiliations:** 1 Vagelos College of Physicians and Surgeons, Columbia University, New York, NY 10032, USA; 2 GE HealthCare, Marlborough, MA 01752, USA; 3 Boston Children’s Hospital, Boston, MA 02115, USA; 4 Department of Pediatrics, University of California, San Francisco, San Francisco, CA 94158, USA; 5 Department of Pediatrics, Baylor College of Medicine, Houston, TX 77030, USA; 6 Department of Molecular and Human Genetics, Baylor College of Medicine, Houston, TX 77030, USA; 7 Lead contact

## Abstract

Cytotoxic immune cells mediate precise attacks against diseased cells to maintain organismal health. Their operational unit of killing and host defense is lytic granules (LGs), which are specialized lysosomal-related organelles. Precision in cytotoxicity is achieved by converging the many LGs to the microtubule-organizing center (MTOC) and polarizing these to the diseased cell for secretion. We identify unappreciated intimate relationships between the Golgi, MTOC, and LGs after cytotoxic cell activation, as well as the *trans*-Golgin protein GCC2 on the LG surface. GCC2 serves to tether LGs to the Golgi following convergence, and both GCC2 and the Golgi are required for the persistence of convergence. GCC2 allows LGs to utilize the Golgi as a docking station preventing LG dispersion and innocent bystander killing in complex three-dimensional environments. We also identify *GCC2* variants causing human natural killer cell deficiency, further emphasizing the importance of LG convergence and Golgi linkage in precision targeting for human immunity.

## In brief

During NK cell activation, lytic granules (LGs) converge to the MTOC, enabling precision in cytotoxicity. Pedroza et al. demonstrate that GCC2 maintains convergence via tethering LGs to the Golgi. Absence of GCC2 leads to LG dispersion, non-directed degranulation, and bystander killing, and biallelic missense variants result in human NK cell deficiency.

## Graphical Abstract

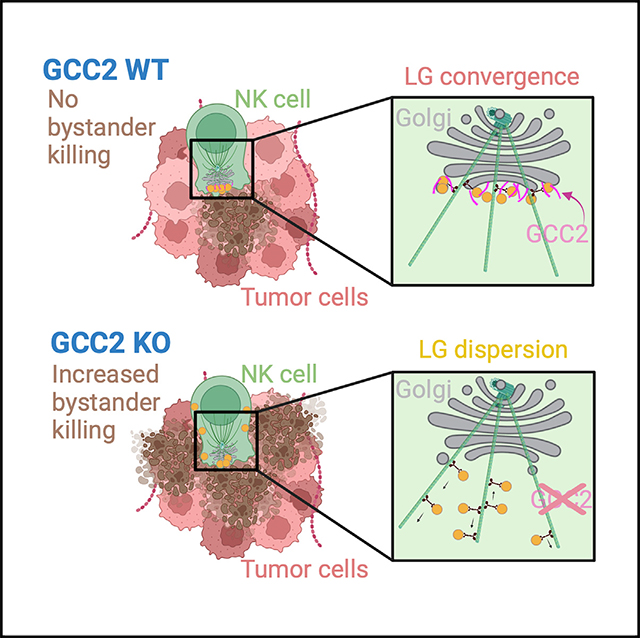

## INTRODUCTION

Natural killer (NK) cells are innate immune cells specialized for the surveillance of malignancy and defense against viral infection and utilize cell-mediated cytotoxicity.^1^ During recognition of diseased cells, NK cells form a lytic immunological synapse (IS) to organize their cytotoxic machinery, precisely eliminating the triggering cell. The lytic IS enables the directed release of perforin and granzymes contained within specialized secretory lysosomal organelles known as lytic granules (LGs), specifically onto the diseased cell.^2^ The release of only one LG is capable of eradicating a targeted cell, although an individual NK cell can contain over a hundred, necessitating precise control of LG positioning and release.^3^ While LGs are scattered throughout the cytoplasm in resting cells, prior to release onto a target, they converge to the microtubule-organizing center (MTOC) and then polarize to the IS.^4^ LG convergence requires dynein motors and utilizes minus-end movement along microtubules.^5^ Once the converged LGs are polarized toward the IS, they are precisely directed onto the target cell. This tightly regulated process promotes efficiency in eradicating the triggering cell and prevents the killing of neighboring healthy cells, known as bystander killing.^2,5^ This “honing activity” is highly desirable for an NK cell’s surveillance functions in which a diseased cell may be amid many that are not targeted for elimination.

Much of the precision in lytic functions of cytotoxic cells after their activation by a diseased cell derives from the control and specific release of the LG. As an organelle with such truly destructive potential, its precise harnessing is critical to cell survival while allowing access to critical host defense. The cytotoxic cell protects itself from the contents of released LGs via membrane lipid ordering, which is reinforced locally by the hyper-ordered LG membrane itself.^6^ The process of LG convergence, however, is critical in ensuring control of these destructive payloads. Many proteins that interact with the LG surface are required for their motility, docking, and ultimately fusing with the synaptic membrane. Some proteins also allow LGs to navigate the dense cortical F-actin meshwork that defines the IS, which can further serve to govern their release. Maintaining a converged LG state, however, would seem to be an efficient stance for an armed cytotoxic cell to take. This process, however, is LG-associated dynein driven, which seems inefficient when considering that the polarization of the MTOC with converged LGs to the IS also requires dynein function.^7^ Importantly, LG convergence can be uncoupled from polarization and does not represent a commitment to killing.^2,7^ LG convergence occurs rapidly and is sustained throughout cytotoxic cell activation. How this occurs, however, and any internal LG “dock” are unclear.

The Golgi complex represents a known source of vesicle biogenesis and sorting. It also exists in proximity to the MTOC under certain circumstances and in immune cells at the IS under others.^8–10^ Here, we augment this observation in NK cells after activation and identified the Golgin family member GCC2 (also known as GCC185) on the surface of the LG as a critical component of the Golgi-LG interaction. We found that GCC2 is required for NK cell LG convergence and promotes their tethering to the Golgi after activation. The loss of GCC2 leads to the dispersion of LGs and non-directional bystander killing. We also identified GCC2 as essential to physiological NK cell function and host defense through its definition as a human NK cell deficiency (NKD) gene. Human biallelic *GCC2* variants block LG convergence and reduce directed killing efficiency, thus linking LG convergence to human host defense.

## RESULTS

### Golgi is associated with LG convergence to the MTOC

**Figure 1.**
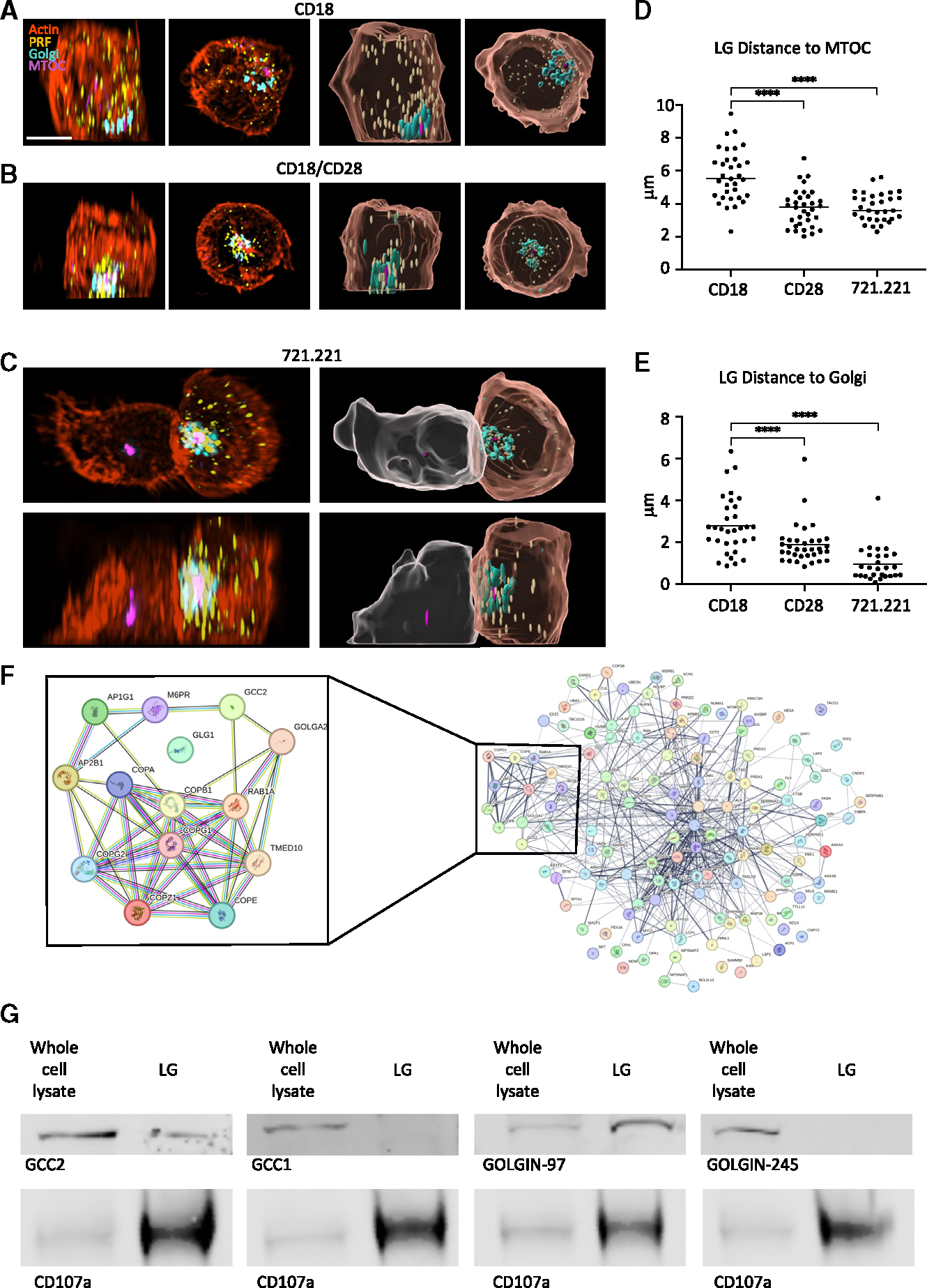
The Golgi associates with lytic granules after NK cell activation (A–C) YTS cells stably transduced with GALT-mCherry allow Golgi visualization (teal) imaged in combination with F-actin (via phalloidin, orange), the MTOC (via α-tubulin foci, purple), and LGs (via perforin, yellow) in cells adhered with non-activating anti-CD18(IB4) (A), cells activated with anti-CD18(IB4)/anti-CD28 (B), or in conjugation with 721.221 target cells (C). Left: original images; right: reconstructions using Imaris software (target cells, gray; scale bar, 10 μm). Left images in each pair show *x*, *z*; right images, *x*, *y* projections. (D and E) Average distance of LGs to the MTOC (D) and the Golgi (E) for 20 to 25 cells per condition: each point represents average distance in one cell derivative from 3 independently repeated experiments (**** = *p* < 0.0001 Mann-Whitney U tests). (F) Visualization of interaction of proteins identified on the LG surface by mass spectrometry after their surface biotinylation and streptavidin-based isolation. Inset denotes known protein associations with Golgi via STRING. (G) Western blot analysis confirmation of *trans*-Golgins within proteins extracted from purified LGs.

Early electron microscopy studies have demonstrated that the Golgi approximates the IS in cytotoxic cells.^8–11^ Given the robust coordination of the lytic machinery involved in cytotoxic host defense function, we considered the potential for the Golgi as a physical regulator of LG positioning and release. To evaluate an interaction between LGs and the Golgi, the YTS human NK cell line was stably transduced with β1,4-galactosyltransferase, fused with the fluorescent protein mCherry (GALT-mCherry).^12^ In resting cells (adhered using non-activating anti-CD18 [IB4]) monoclonal antibody-coated glass) (Figure S1A), LGs were scattered in the cytoplasm having no clear association with the Golgi or the MTOC despite the Golgi and MTOC being in close proximity to each other (Figure 1A). However, after stimulation with an anti-CD18(IB4)/anti-CD28-coated surface (Figure 1B), or 721.221 target cells (Figure 1C), the LG converged to the MTOC (Figure 1D) and occupied the same space as the Golgi (Figure 1E), raising the possibility of a role for the Golgi in LG function.

To investigate the possibility of a physical interaction between LGs and the Golgi, LGs were purified from YTS cells, and their surface proteins were biotinylated and isolated using streptavidin-coated magnetic beads as previously described.^13^ This isolated set of LG surface proteins was then sequenced using mass spectrometry (Figure 1F; Table S1), and we focused on those known to associate with the Golgi (Figure 1F inset). These included GCC2, known to be involved in retrograde transport of vesicles,^14–16^ which is part of a family of *trans*-Golgi proteins that includes GCC1, GOLGA1 (Golgin-97), and GOLGA4 (Golgin-245).^17^ GCC2 has no known cargoes.^18^

Although GCC2 is a known *trans*-Golgi protein, its interaction with Rab6A and ARL1 leads to its Golgi association being indirect and transient, allowing it to switch between different compartments.^14^ A proposed model is that cytosolic GCC2 interacts with vesicles on microtubules that subsequently tether to the Golgi.^14^ To confirm the presence of GCC2 and other Golgins on LGs independently of the Golgi, western blot analysis was performed using lysates from purified LGs compared to whole-cell lysates. The presence of CD107a was used as a control and was enriched in LGs as expected. The four *trans*-Golgin proteins GCC2, GCC1, Golgin-97, and Golgin-245 were evaluated, and while all were found in NK cells, only GCC2 and Golgin-97 were identified with LGs (Figure 1G). To ensure the lack of Golgi contamination in the isolated LG fraction, western blot analysis for giantin, a *cis*-Golgi protein, was performed and was absent (Figure S1B). Collectively, these data demonstrate the presence of select *trans*-Golgi resident proteins with LGs and raise the question of whether they possess any functional relevance.

### GCC2 tethers LGs during activation-induced convergence

**Figure 2.**
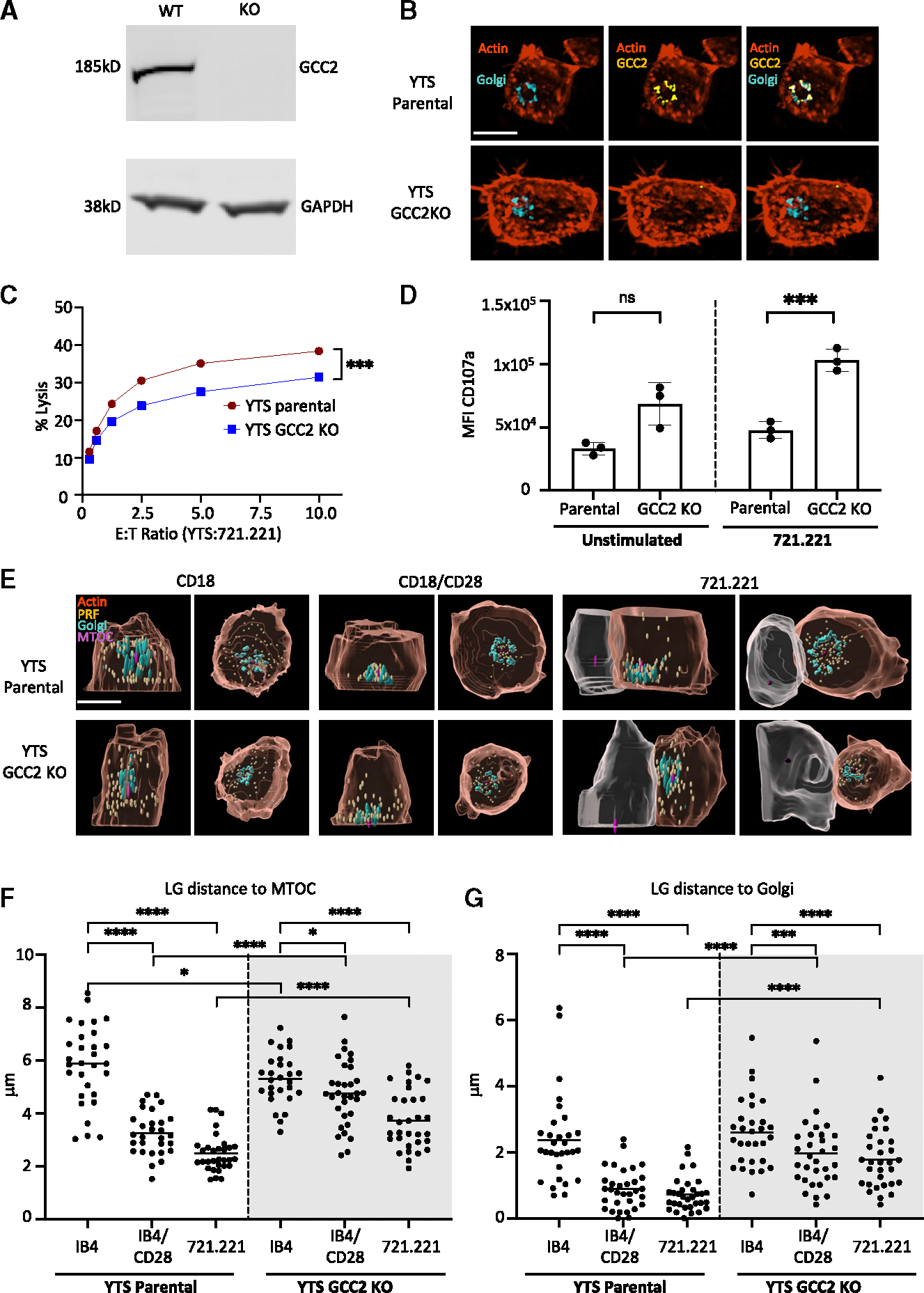
GCC2 is required for activation-induced LG convergence (A and B) GCC2 expression was confirmed by (A) western blot analysis of whole-cell lysate and (B) confocal microscopy: F-actin (phalloidin, orange), Golgi (GALT-mCherry, teal), and GCC2 (anti-GCC2, yellow); scale bar, 10 μm. (C) Representative 4 h ^51^Cr-release assay cytotoxicity using YTS GCC2 KO or parental cells against 721.221 target cells (*** = *p* < 0.001 chi-squared tests). (D) Degranulation in YTS GCC2 KO or parental cells after 60 min of incubation with 721.221 target cells measured by mean fluorescence intensity (MFI) of CD107a. Data represented as mean ± SD of 3 independent repeats with individual values depicted (* = *p* < 0.05, *** = *p* < 0.001 Mann-Whitney U tests). (E) Representative rendered images from YTS parental or GCC2 KO cells stimulated with anti-CD18(IB4)/anti-CD28-coated glass or 721.221 target cells for 60 min: LGs (perforin, yellow), F-actin (phalloidin, orange), Golgi (mCherry-GALT, teal), and MTOC (α-tubulin purple); scale bar, 10 μm. The left image in each pair shows an *x*, *z* and the right image an *x*, *y* projection. (F and G) Mean distance of LGs to MTOC (F) and Golgi (G) measured from 20 to 25 cells per condition across 3 independent experiments in YTS parental (left) and YTS GCC2 KO (right, shaded) cells. (noted comparisions were different **** = *p* < 0.0001, *** = *p* < 0.001, * = *p* < 0.05, Mann-Whitney U tests).

A GCC2-deficient cell line was generated in YTS cells using CRISPR-Cas9 editing (YTS GCC2 knockout [KO]) in order to determine the role of GCC2 in NK cells, and the deletion of GCC2 was confirmed by western blot (Figure 2A) and confocal microscopy (Figure 2B). YTS cells are clonal and have a large number of LGs that are measurably converged after activation and prior to polarization to the IS.^7,19^ This parallels *ex vivo* NK cell biology and makes YTS cells suitable for the study of LG dynamics. The deletion of GCC2 in YTS cells did not affect the gross morphology of the Golgi (Figure 2B). Their function in directed cytotoxicity against a transformed target cell was overall maintained albeit slightly reduced as measured using a ^51^Cr-release assay (Figure 2C). To evaluate for a potential explanation for this reduction, the ability of YTS GCC2 KO cells to degranulate after stimulation was measured by flow cytometry using CD107a as a measure of granule release. Interestingly, however, the GCC2-deficient cells demonstrated increased and not decreased degranulation after activation (Figure 2D). This was confirmed using cells expressing a fluorescence-based degranulation indicator (mApple-LAMP1-pHluorin) and measuring the number of degranulation events (pHluorin fluorescence transitions)^3^ on an activating anti-CD18(IB4)/anti-CD28-coated surface (Figure S1D). Thus, directed cytotoxicity in the absence of GCC2 was decreased, but with increased degranulation.

To explain this unusual combination of behaviors, we considered a decreased efficiency of directed secretion for cytotoxicity that might allow for more degranulation with less targeted killing. Although increased degranulation could suggest a direct role of GCC2 in LG exocytosis, its primary expression on the Golgi suggested a role in earlier events when LGs approach the Golgi, specifically convergence. Given that convergence is a major driver of LG positioning and efficiency in directed synaptic degranulation, we measured convergence to the MTOC in GCC2 KO and parental YTS stimulated on an anti-CD18(IB4)/anti-CD28-activating surface or with 721.221 target cells. Following stimulation, LG convergence was incomplete in YTS GCC2 KO cells, with many remaining dispersed relative to the MTOC (Figures 2E and 2F) or the Golgi (Figures 2E and 2G). Thus, GCC2 was required for obtaining activation-induced LG convergence and approximation to both the MTOC and the Golgi.

**Figure 3.**
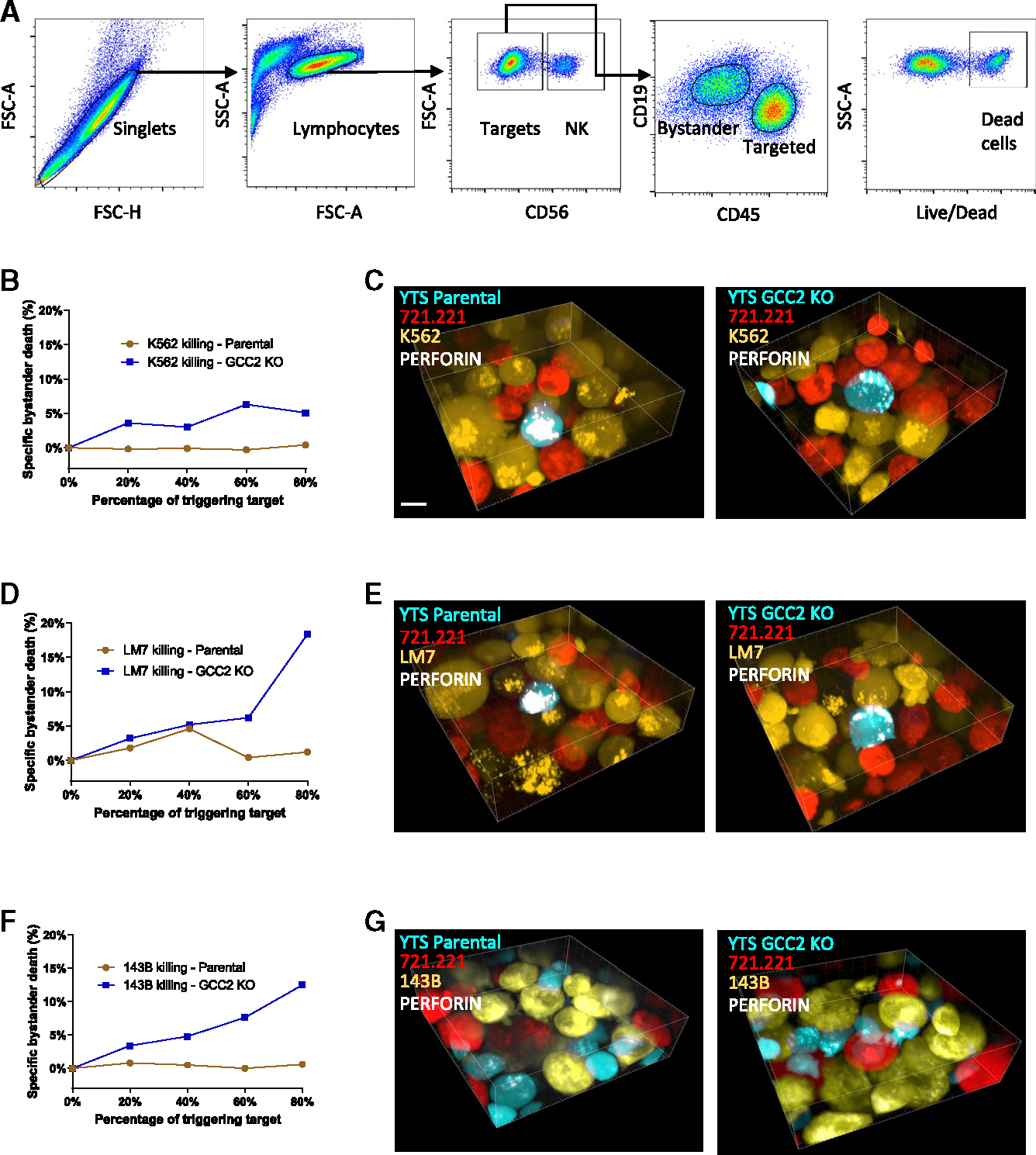
GCC2 prevents bystander killing in multicellular microenvironments (A) TheCOS stacks were digested, and cells were stained using LIVE/DEAD Near-IR and anti-CD56, -CD45, and -CD19 to allow for identification of live and dead effector, target, and bystander cells via gating. (B) Representative results of resistant K562 target cell “bystander” killing in TheCOS via flow cytometry by parental YTS (red) or YTS GCC2 KO (blue) with increasing percentages of susceptible 721.221 cells from 0 to 80% of the target cells in the TheCOS stack. (C) TheCOS stacks were cut and placed upon glass and imaged via confocal microscopy through the z axis. Three-dimensional reconstruction of images were generated to show vital dye-labeled parental YTS (teal), 721.221 triggering target cells (red), and resistant K562 “bystander” cells (yellow) with staining for perforin to visualize LGs (white); scale bar, 10 μm. (D–G) TheCOS experiments using the osteosarcoma cell lines LM7 (D and E) or 143B (F and G) substituted for the K562 “bystander” cells. After digestion of TheCOS stacks, (D) cells were evaluated by flow cytometry to measure LM7 osteosarcoma cell “bystander” killing by parental YTS (red) or YTS GCC2 KO (blue) cells with increasing percentages of susceptible 721.221 cells ranging from 0 to 80%. (E) Three-dimensional reconstruction of a TheCOS stack: vital dye-labeled parental YTS (teal), 721.221 triggering target cells (red), resistant LM7 osteosarcoma “bystander” cells (yellow), and perforin for LGs (white); scale bar, 10 μm. (F) After digestion of a TheCOS stack, cells were evaluated by flow cytometry to measure the 143B osteosarcoma cell “bystander” killing mediated by parental YTS (red) or YTS GCC2 KO (blue) cells with increasing percentages of susceptible 721.221 cells ranging from 0 to 80%. (G) Three-dimensional reconstruction of a TheCOS stack: vital dye-labeled parental YTS (teal), 721.221 triggering target cells (red), resistant 143B osteosarcoma “bystander” cells (yellow), and perforin for LGs (white); scale bar, 10 μm.

One function of LG convergence is to prevent non-directional LG release and bystander cell killing. Thus, we next wanted to determine how the dispersed LGs in GCC2 KO cells function in complex three-dimensional environments containing both targeted and non-targeted cells. We utilized a thermal collapse of strata (TheCOS) system to approximate different types of cells in specified ratios, quickly upon demand via utilizing cells embedded in layers of temperature-sensitive hydrogels.^20^ Using TheCOS, YTS parental or GCC2 KO cells embedded in layers could be approximated to triggering 721.221 target cells and non-targeted K562 cells at the same time after the alteration of ambient temperature. To assess the function of the YTS cells against the different 721.221 and K562 cells, a TheCOS stack was digested after 4 h, and individual cells were isolated, stained, and fixed for flow cytometric analysis (Figure 3A). This allows for the identification of the different types of cells after mixing via TheCOS as well as their viability using nuclear dye. As expected, the parental YTS cells were unable to kill the resistant K562 “bystander cells” even in the presence of a majority of 721.221-triggering target cells (Figure 3B, red). When GCC2 KO cells were evaluated, however, killing of K562 cells was apparent, and increased with increasing percentages of triggering 721.221 cells (Figure 3B, blue). In parallel and from the same experiment, a slice of an undigested TheCOS gel stack was evaluated using confocal microscopy and demonstrated the intimate contact of the YTS cells with both the 721.221 and K562 cells (Figure 3C). Furthermore, the LGs, as denoted by Lysotracker staining, were converged in the parental YTS cells but dispersed in the YTS GCC2 KO cells, demonstrating that the convergence and dispersion of LGs were maintained in the NK cells even when in a complex three-dimensional environment. Importantly, the YTS GCC2 KO cells with dispersed LGs were able to mediate off-target killing in a three-dimensional environment and promote the destruction of a bystander cell.

To evaluate the impact of GCC2-directed LG convergence in a setting more relevant to a human solid tumor, we performed similar experiments using human osteosarcoma cell lines, including the highly resistant LM7 and the relatively resistant 143B tumor cells. Neither YTS nor GCC2 KO cells demonstrated measurable killing of either 143B or LM7 cells in the absence of a triggering target using TheCOS, and thus both were resistant (Figures 3D, 3F, and S1E for ^51^Cr killing assay). When triggering 721.221 target cells were added, however, the killing of osteosarcoma cells was detectable, but only by the GCC2 KO cells. This “bystander” killing effect against the 143B and LM7 cells increased with increasing percentages of triggering 721.221 target cells present in TheCOS. As an example of the complex cell arrangement between YTS, 721.221, and osteosarcoma cells, undigested TheCOS stack slices from the highly resistant LM7 and 143B cell experiments were visualized using confocal microscopy (Figures 3E and 3F). In experiments using either the YTS parental or GCC2 KO cells, these cells could be seen in close approximation with a 721.221 target cell and osteosarcoma cells. The LGs in the parental YTS cell were converged, but those in the GCC2 KO were dispersed. Thus, LG dispersion owing to the absence of GCC2 was associated with increased bystander cell killing after triggering even when these bystander cells were otherwise resistant human osteosarcoma cells. This suggests that GCC2 regulates LG convergence to promote efficiency against a triggering target cell and prevents killing of neighboring cells in a simulated three-dimensional tumor microenvironment setting.

### Ectopic expression of GCC2 at the cell membrane redirects LGs to the cell surface after activation

**Figure 4.**
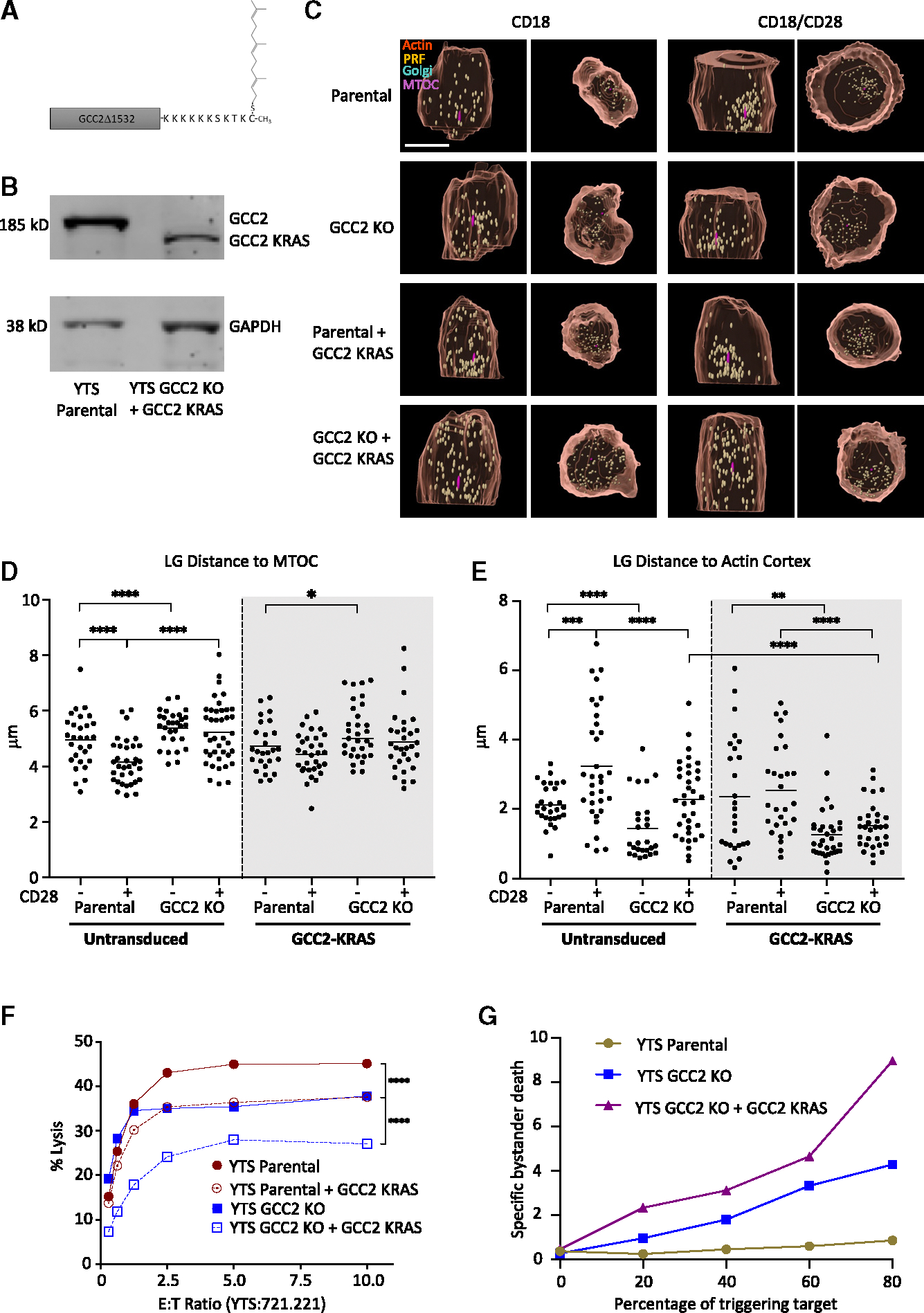
Ectopic expression of GCC2 at the cell membrane blocks LG convergence and enhances bystander killing (A) Construct used for targeting GCC2 to the cell membrane. (B) Western blot analysis for GCC2 of whole-cell lysates confirming GCC2-KRAS expression (178 kDa) vs. endogenous GCC2 (185 kDa). (C) Representative reconstructed YTS cell images from parental and GCC2 KO, or GCC2 KO YTS reconstituted with the GCC2-KRAS, after stimulation with anti-CD18(IB4)/anti-CD28-coated glass for 60 min. LGs (perforin, yellow), F-actin (phalloidin, orange), Golgi (GALT-mCherry, teal), and the MTOC (α-tubulin purple); scale bar, 10 μm, *x*, *z* (left) and *x*, *y* (right) projections. (D and E) Mean distance of LGs to MTOC (D) and to the F-actin cortex (E) measured from 20 to 25 cells per condition across 2 independent experiments in YTS parental and GCC2 KO (left) and when stably transduced with GCC2-KRAS cells (right, shadowed). (noted comparisions were different **** = *p* < 0.0001, *** = *p* < 0.001, ** = *p* < 0.01, * = *p* < 0.05, Mann-Whitney U tests). (F) Cytotoxicity of YTS or GCC2 KO YTS cells with or without the GCC2-KRAS construct against 721.221 target cells measured by ^51^Cr-release assay. Individual points show technical triplicate means and represent 3 independent repeats (*** = *p* < 0.001 chi-squared test). (G) Representative flow cytometry viability assays after 4 h killing in TheCOS. Resistant K562 target cell “bystander” killing by parental YTS (brown), YTS GCC2 KO (blue), or GCC2 KO YTS+GCC2 KRAS (purple) with increasing percentages of susceptible 721.221 cells from 0 to 80% of the target cells in the TheCOS stack.

Since endogenous GCC2 appeared to link LGs to the Golgi during convergence, we wanted to determine if altering the cellular localization of the protein could redirect LG positioning. GCC2 localization is directed by its GRIP domain that is responsible for its Golgi anchoring, and replacing this with a monoamine oxidase A sequence has been shown to divert it to mitochondria.^21^ Since under some circumstances LGs can interact with mitochondria,^22^ we developed a different localization strategy and replaced the C terminus of the GCC2 GRIP domain with the last 14 residues of the KRAS protein^23^ (Figure 4A). This residue is known to be farnesylated, which should promote cytoplasmic membrane interaction. When YTS GCC2 KO cells were stably transduced with the GCC2 1532-C14KRAS construct (GCC2-KRAS), the fusion protein deleted of the C-terminal 104 residues could be identified by western blot analysis of total lysates (Figure 4B). We next stimulated YTS parental and GCC2 KO GCC2-KRAS cells to evaluate if the localization of LGs was altered relative to the MTOC and cell surface in the presence of the GCC2 fusion protein. We used the F-actin cortex labeled via phalloidin as a surrogate for the cell membrane owing to its proximity to it and its robustness in fixed cells. As expected, activation via CD28 induced LG convergence to the MTOC in parental cells, but not GCC2 KO cells, irrespective of the presence of the GCC2-KRAS construct (Figures 4C and 4D). When the GCC2-KRAS construct was present, however, the LGs were found closer to the cell cortex after CD28 activation, with increased significance in the absence of endogenous GCC2 (Figures 4C and 4E). Thus, ectopic localization of GCC2 promoted mislocalization of LGs toward targeted sites of GCC2 expression.

To evaluate the effect of forced LG localization, directed cytotoxic activity was measured in the presence of the GCC2-KRAS construct. The expression of GCC2-KRAS in parental YTS cells reduced their killing efficiency against 721.221 target cells to the level of GCC2 KO cells (Figure 4F). Directed killing efficiency was reduced further still in GCC2 KO cells containing GCC2-KRAS. This construct, however, did not impair the ability of GCC2 to interact with LGs as isolated LGs from these cells could still be found to contain the GCC2-KRAS construct (Figure S4D). Given that the LGs in the GCC2-KRAS-expressing cells were dispersed and more peripherally localized after activation, we wanted to evaluate if there was an impact upon bystander killing of neighboring non-targeted cells after NK cell triggering. Thus, we measured the killing of non-targeted K562 cells using TheCOS when increasing percentages of triggering 721.221 cells were present. As triggering target cells were added, there was increased measurable specific killing of K562 bystanders in the absence of GCC2, but not in parental YTS cells (Figure 4G). This specific bystander killing in the GCC2 KO cells was increased further still by the addition of GCC2-KRAS. While this may relate to the level of GCC2-KRAS expression, it suggests that convergence to the MTOC and Golgi dominates in preventing bystander killing. Conversely, redirection of LGs to the cell membrane results in reduced IS-mediated killing and increased non-IS-mediated killing.

### The Golgi complex modulates LG positioning during convergence

**Figure 5.**
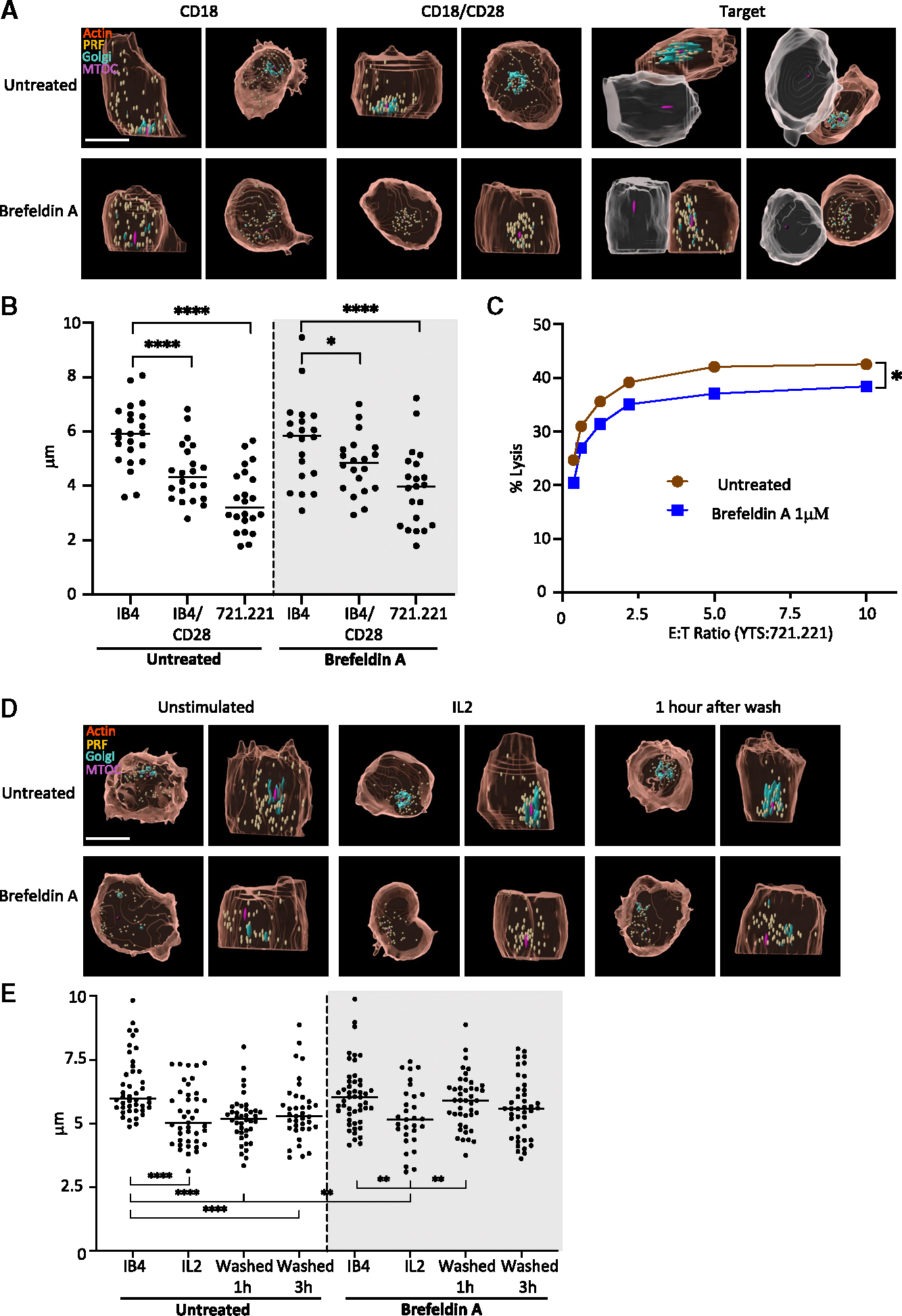
Golgi maintains LG convergence after activation (A) Representative rendered images of YTS cells untreated or treated with 1 μM Brefeldin A and stimulated with anti-CD18(IB4)/anti-CD28-coated glass or by 721.221 target cells for 60 min. LGs (perforin, yellow), F-actin (phalloidin, orange), Golgi (GALT-mCherry, teal), and the MTOC (α-tubulin purple), and target cells (gray), scale bar, 10 μm, *x*, *z* (left) and *x*, *y* (right) projections. (B) Mean distance of LGs to the MTOC in 20–25 cells per condition across 3 independent experiments in YTS that were unstimulated (anti-CD18 [IB4]) or stimulated (anti-CD18 [IB4]/anti-CD28) on coated glass or by conjugation with 721.221 target cells for 60 min; pretreated with (right, shadowed) or without (left) 1 μM Brefeldin A (noted comparisons were different **** = *p* < 0.0001, * = *p* < 0.05, Mann-Whitney U tests). (C) Representative 4 h ^51^Cr release killing assay against 721.221 target cells using YTS cells untreated (red) or pretreated (blue) with 1 μM Brefeldin A (means of technical triplicates, representative assay of *n* = 3, *p < 0.05 chi-squared tests). (D) Rendered images of YTS cells untreated (top) or treated with 1 μM Brefeldin A (bottom); unstimulated (left), stimulated with 100 units/mL IL-2 for 30 min (center), or stimulated for 30 min followed by IL-2 washout and 1 h incubation at 37°C (right). LGs (perforin, yellow), F-actin (phalloidin, orange), Golgi (GALT-mCherry, teal), and MTOC (α-tubulin, purple), *x*, *z* (left) and the right image *x*, *y* (right) projections. (E) Mean distance of LGs to the MTOC from 20 to 25 cells per condition (2 independent experiments) in untreated (left) or Brefeldin-treated (right, shadowed) cells after IL-2 stimulation, and 1 or 3 h after washing the stimulus. (noted comparisons were different *** = *p* < 0.0001, ** = *p* < 0.01, * = *p* < 0.05 Mann-Whitney U tests).

Since the Golgi-related protein GCC2 appears essential for at least part of LG convergence after NK cell activation, we wanted to determine any direct role of the Golgi in LG positioning. To eliminate the Golgi as an MTOC proximal presence, the Golgi was fragmented using Brefeldin A prior to and during NK cell stimulation. In Brefeldin A-treated cells, the Golgi as labeled by mCherry-GALT were no longer aggregated around the MTOC in either resting or activated cells (Figure 5A). Surprisingly, the fragmentation of the Golgi did not grossly impact NK cell LG convergence after their activation on anti-CD18(IB4)/anti-CD28-coated glass or when in conjugation with 721.221 target cells. Detailed measurements of convergence after activation confirmed no significant alteration of LG positioning relative to the MTOC (Figure 5B). When fragmented, however, the proximity of Golgi fragments to LGs was still maintained, but only when GCC2 was present (Figures S2A–S2B). The specificity of LG positioning was further considered in this context by contrasting their localization to that of mitochondria, which were unaffected by either NK cell activation or Golgi fragmentation (Figures S2C–S2D). Finally, killing efficiency as determined by ^51^Cr-release assay was only minimally affected by Brefeldin treatment and Golgi fragmentation (Figures 5A–5C). Thus, the positioning of the Golgi appears to be dispensable for LG convergence to the MTOC and resulting directed cytotoxicity.

Given that GCC2 was required for full LG convergence to the MTOC after activation and LGs to Golgi approximation, we instead considered a role for the Golgi in sustaining LG convergence. To test this, we induced LG convergence using soluble interleukin-2 (IL-2) that promotes LG movement to the MTOC dependent upon non-canonical signaling via Src family kinase-mediated convergence.^4^ IL-2 added to media induced significant LG convergence to the MTOC in YTS cells (Figures 5D and 5E), albeit at levels lower than those measured after target cell engagement. The disaggregation of the Golgi using Brefeldin A treatment did not affect the IL-2-induced convergence of LG, consistent with the observation in glass surface or target cell-activated NK cells. We next removed the IL-2 convergence signal after IL-2-induced NK cell activation by washing the cells and placing them into media without IL-2. Despite the removal of IL-2, there was sustained convergence of LGs in otherwise untreated NK cells after 3 h (Figures 5D and 5E). Importantly, however, in the presence of Brefeldin A, the removal of IL-2 resulted in a dispersion of LGs after 1 h. Thus, when the Golgi were fragmented and not MTOC localized, LG convergence was not sustained. This suggests that the LGs use tethering to the Golgi as a means for controlling their positioning and maintaining a converged state after having experienced a convergence signal. This, in turn, promotes maximal efficiency in directed cytotoxicity and prevents bystander killing.

### Biallelic GCC2 mutations cause human NK cell deficiency

**Figure 6.**
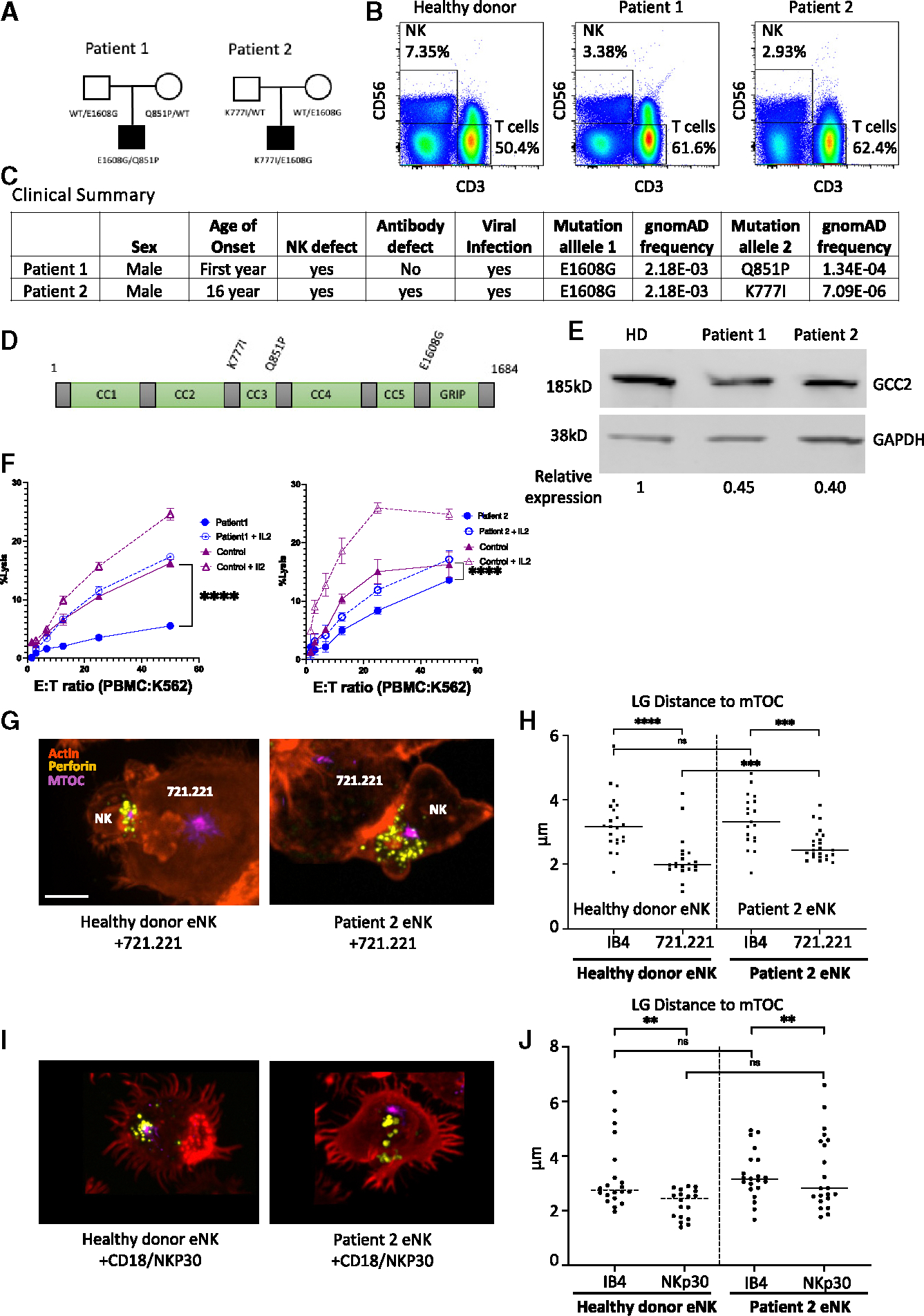
Biallelic mutations in GCC2 lead to an NK cell deficiency with impaired LG convergence (A) Patients pedigree with biallelic *GCC2* variants. (B) Flow cytometric analysis of total NK cells among PBMC. (C) Clinical and variant summary. (D) Schematic GCC2 with interspaced CC-D and GRIP domain regions (green) and disordered regions (gray) showing variant localization. (E) Western blot analysis of GCC2 expression in PBMC whole-cell lysates (HD relative expression shown at the bottom). (F) 4 h ^51^Cr-release assay of PBMC (patients, blue; HD purple) against K562 targets with (dashed line) or without (solid line) 1,000 U/mL added IL-2 (points = means of technical triplicates; representative assay of *n* = 2 is shown; **** = *p* < 0.0001 chi-squared test). (G) Confocal microscopy (*x*, *y* plane) of isolated NK cells from patient 2 incubated 60 min with 721.221 targets showing F-actin (phalloidin, orange), LGs (perforin, yellow), and MTOC (α-tubulin, purple); scale bar, 10 μm. (H) Mean distance of LGs to the MTOC after adherence to non-activating (anti-CD18 [IB4]) glass surface or conjugated to 721.221 target cells; minimum 20 cells per condition (noted comparisons were different **** = *p* < 0.0001, *** = *p* < 0.001, or not significant [ns] Mann-Whitney U tests). (I) Confocal microscopy (*x*, *y* plane) of isolated NK cells from patient 2 incubated 60 min on glass coated with anti-CD18(IB4) and anti-NKp30 showing F-actin (phalloidin, orange), LGs (perforin, yellow), and MTOC (α-tubulin, purple); scale bar, 10 μm. (J) Mean distance of LGs to the MTOC from a minimum of 20 cells per condition (noted comparisons were different; **** = *p* < 0.0001, *** = *p* < 0.001, ** = *p* < 0.01, or not significant (ns), Mann-Whitney U tests).

Since directed cytotoxicity as enabled by LG convergence and polarization is a central theme in killing efficiency, we suspected that interference with these functions could impair the ability of NK cells to serve typical human host defense. Since removal of GCC2 reduced the convergence function in NK cells without preventing their ability to divide and proliferate, we considered *GCC2* as a candidate gene for causing human NKD. NKD is defined as a stable deficiency of NK cell number or function that represents an individual’s major clinical immunodeficiency, mostly leading to susceptibility to viral infections and cancers.^24,25^ We have been evaluating individuals suspected of having NKD since 2006 and have performed exome sequencing on many of them. Thus, we queried our unsolved cases for variants in *GCC2* or related genes and identified two unrelated individuals with compound heterozygous biallelic variants in *GCC2* (Figure 6A). Both patient percentages of total NK cells in the peripheral blood were within normal limits (Figure 6B). The two individuals presented in childhood (age 1 and age 16) had a history of recurrent viral infection and lacked known immunodeficiency gene variation (Figure 6C and Methods S1).

Interestingly, both individuals shared a single GCC2 variant E1608G in combination with a second Q851P in patient 1 and K777I in patient 2 (Figure 6D). The maximal estimated combinatorial incidence of these variants would be 2 in 10 million according to gnomAD.^26^ GCC2 has 1,684 residues and contains 5 coiled-coil domains (CC-Ds) and a C-terminal GRIP domain all separated by disordered regions (Figure 6D). The shared E1608G variant is at the beginning of the GRIP domain, and the other two are in or near the third CC-D, and *in silico* modeling using AlphaFold3^27,28^ predicts that Q851P, but not K777I, and E1608G are within a CC-D region (Figures S3A–S3C). Interestingly, all three variants lie within proximity of known Rab-interacting regions, Rab9 (residues 805–889) and Rab6a (residues 1,609–1,659).^29^ To determine if there was expression of GCC2 protein in the setting of these biallelic variants, peripheral blood mononuclear cells (PBMCs) from patients 1 and 2 were used for western blot analysis (Figure 6E), and GCC2 was detectable in both, albeit at somewhat lower levels than those found in a set of healthy control donors (Figures 6E and S4A). Finally, to confirm the expression pattern of the patient GCC2 variants, microscopy was performed using patient NK cells. GCC2 demonstrated a Golgi pattern of expression in both patient and control cells suggesting that the variant GCC2 protein expressed in patient NK cells was not impaired in localization (Figure S4B.).

To determine the potential effects of these GCC2 variants in NK cells from patients, patient samples were evaluated directly, via ^51^Cr-release assay against K562 target cells, and demonstrated reduced killing in both patients (Figure 6F). A substantive portion of killing could be restored by short-term stimulation with IL-2, but not to the levels of stimulated healthy control donor cells. Although the percentage of NK cells in the patients was normal, we evaluated the maturity and developmental trajectory of their peripheral NK cells in detail by high-parameter flow cytometry. The relative developmental subset frequency and phenotype found in the patient NK cells was generally indistinguishable from healthy control donor cells (Figure S5). This suggests that the defect associated with biallelic *GCC2* variants was functional rather than developmental (unlike most known NKD). Therefore, we next evaluated LG positioning in purified *ex vivo* patient NK cells conjugated with 721.221 target cells or an activating surface with anti-NKp30. After 1 h, both healthy donor and patient NK cells formed lytic immune synapses with LGs converged to the MTOC (Figure 6G). LG convergence measured across multiple synapses, however, was significantly reduced in patient NK cells (Figure 6H). Thus, the impact of biallelic GCC2 variants in NK cells from patients with NKD appeared similar to that of GCC2 KO YTS cells with reduced directed cytotoxicity and LG convergence.

**Figure 7.**
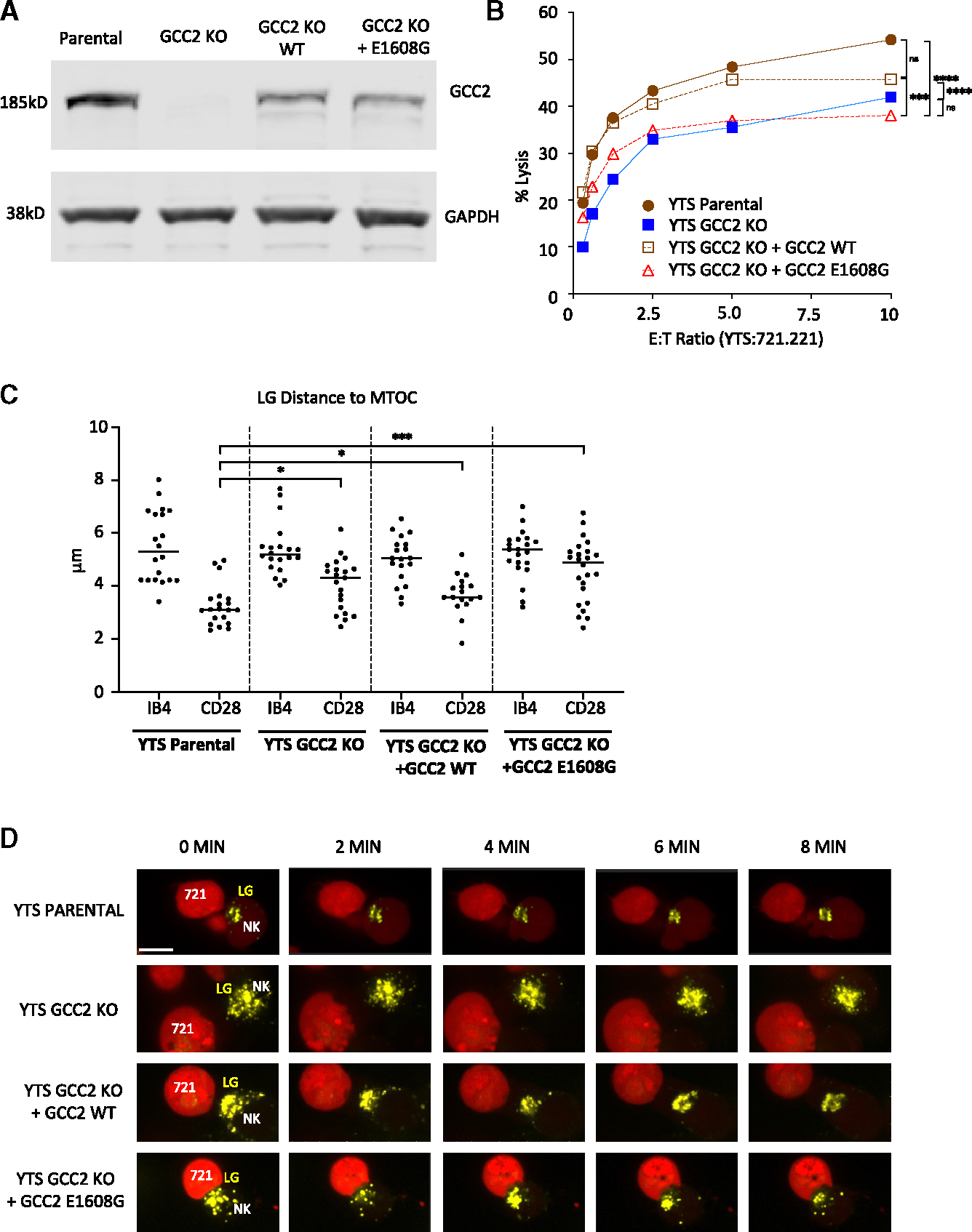
The common NKD GCC2 variant impairs directed killing and LG convergence (A) GCC2 reconstitution confirmed by western blot analysis of whole-cell lysates. (B) 4 h ^51^Cr-release assay against 721.221 targets by YTS GCC2 KO cells reconstituted with either WT GCC2 or GCC2 E1608G. Individual points show technical triplicate means, showing a representative assay of *n* = 3 (noted comparisons were different **** = *p* < 0.0001, *** = *p* < 0.001, chi-squared test). (C) Mean distance of LGs to the MTOC in YTS GCC2 KO cells reconstituted with GCC2 WT, or GCC2 E1608G (after 40 min of stimulation on IB4/anti-CD28-coated glass; 20–25 cells per condition from 2 independent experiments; noted comparisons were different, *** = *p* < 0.001, * = *p* < 0.05). (D) Time-lapse live-cell confocal microscopy of conjugates between 721.221 targets (red) and YTS parental, YTS GCC2 KO, and YTS GCC2 KO reconstituted with WT GCC2 or GCC2 E1608G pre-loaded with Lysotracker Deep Red, LGs (yellow) (scale bar, 20 μm). Imaging began after 30 min of conjugation (time = 0), and images collected every 5 s (total = 100 frames, images from every ~2 min shown).

To identify the impact of the common GCC2 variant in patients with NKD on NK cell biology, it was evaluated directly in YTS cells (thus, independently of any patient genetic background). YTS GCC2 KO cells were reconstituted with the shared E1608G variant or wild-type (WT) GCC2. Levels of GCC2 protein reconstituted in stably transduced YTS GCC2 KO cells measured by western blot analysis were less than in parental cells but similar between the WT and E1608G variant (Figure 7A). Expression of the WT GCC2 restored the cytotoxicity of GCC2 KO cells to near parental cell levels (Figure 7B). Expression of the E1608G variant, however, failed to increase cytotoxicity relative to those lacking GCC2 altogether. This same trend was also observed in YTS cells transduced with either of the other two patient-derived GCC2 variants (Figure S4C). In order to determine if the interaction of GCC2 variants with LGs was preserved, LGs from the reconstituted cells were isolated and evaluated for the presence of GCC2 by western blot analysis. The common E1608G and Q851P variants retained the ability to interact with LGs, while the K777I had reduced interaction (Figure S4D).

Finally, we evaluated the effect of the common patient variant on LG convergence when used to reconstitute GCC2 KO cells in direct imaging of lytic synapses. The expression of E1608G failed to restore LG convergence after lytic synapse formation (Figure 7C). Time-lapse imaging demonstrated that even the typical convergence found after activation was greatly reduced in the absence of GCC2 and largely eliminated in the presence of the common patient variant (Figure 7D). Thus, GCC2 E1608G was unable to rescue NK cell function and resulted in dispersed LGs after activation. Variant GCC2 functions as an NKD-causing gene by eradicating LG convergence and underscoring that an LG Golgi linkage is needed for optimal LG convergence, directed killing, and human host defense.

## DISCUSSION

One of the main functions of NK cells is to kill virus-infected or malignant cells in a complex context where those cells are usually surrounded by healthy cells. As such, they are hypothesized to be important for immunological surveillance against cancer and other nascent threats.^30,31^ This selectivity is dictated by a complex system of activating and inhibitory receptors engaged in the context of the IS. This in turn triggers intracellular pathways to ensure that LGs containing perforin and granzymes are only released at the activating IS with the targeted cell rather than toward bystander cells.^2,32^ This directionality and specificity is dependent upon LG convergence and subsequent polarization.^2^ While the LG convergence process is mediated by dynein/kinesin-dependent trafficking on microtubules,^2,7^ how this convergence is sustained after activation and throughout polarization until LGs access the F-actin cortex during degranulation is unknown. Given that dynein motors use substantive energy, it would seem logical that there is another mechanism for maintaining convergence after the initial process has occurred. In the context of NK cells, this could represent long periods of time as might be required by an NK cell that has extravasated via a convergence-inducing integrin signal and is in the midst of mediating cytotoxic function within a tissue. It would essentially allow them to persist in an “armed” state.

In the context of restricted intracellular space including numerous organelles having distinct functions, interactions between these organelles are expected and have been studied, such as the interaction of LGs with the centriole^5,7^ or mitochondria^22^ in NK cells. While the Golgi complex is known to be relevant to LG biogenesis,^33^ only incidental data in cytotoxic cells using electron microscopy have observed proximity of the Golgi to LGs and any association of the Golgi with the IS.^8–11^ Primarily known for its role in the maturation and secretion of proteins and vesicles, the Golgi is also critical to endosomal recycling mediated by Golgi membrane proteins that tether and promote fusion.^12,17,29,34^ A first tethering step is mediated in part by the *trans*-Golgins, a family of structurally conserved CC-D proteins that can interact with different vesicles and cargoes.^17^ Generally, *trans*-Golgins have an N-terminal region that interacts with the cargo, a long CC-D region with several Rab interacting domains,^14^ and a highly conserved C-terminal region (GRIP domain) important for Golgi interaction. Of the four *trans*-Golgins, cargo has been described for three, but not GCC2.^18,21^

Since we identified the LGs as converged around the MTOC in the vicinity of the Golgi after NK cell activation, we looked for proteins at the LG surface that could potentially facilitate interaction. We expected to find Rab proteins, which are known to enable Golgi interaction in recycling,^35^ but were surprised to find actual Golgi proteins. Although GCC2 is clearly a Golgi protein owing to its structure, its interaction with the Golgi through the GRIP domain could be considered dynamic due to its dependence on other proteins such as Rab6 and Arl1.^14,15,29^ While GCC2 could be acquired from LG biogenesis, it has been suggested that GCC2 is also cytoplasmic and can interact with vesicles to drive them to the Golgi.^14^ Similar behavior has been suggested for another CC domain protein, HkRP3, in NK cells.^36^ Interestingly, our preliminary unpublished proteomic data suggest an interaction of GCC2 and HkRP3, and thus a model where HkRP3 promotes LG convergence via microtubules, while GCC2 enables tethering at the Golgi, is plausible. Alternatively, GCC2 has been reported as a microtubule-nucleating factor on the Golgi, via CLASP1 interaction,^37^ and could help generate noncentrosomal microtubule fibers that could facilitate the recruitment of LGs to the Golgi. Along these lines, cytotoxic T cells lacking centrioles have reduced, but not absent, MTOC function, suggesting that other nucleating centers such as those formed on the Golgi can function as rudimentary MTOCs.^38^

How GCC2 interacts with LG remains elusive. GCC2 has several Rab-interacting domains,^29^ and Rabs are enriched on the LG surface.^35^ We have attempted to knock down the expression of the 2 main Rab proteins known to interact with GCC2: Rab9A and Rab6A, but found no clear phenotype (not shown). Interestingly, reduction of Rab6A decreased GCC2 presence on the Golgi and increased the LG convergence, similar to the results obtained in Brefeldin-treated cells (FigureS6). While not conclusive, the lack of a clear phenotype with reducing expression of individual Rab proteins suggests a functional role for multiple Rab-GCC2 interactions and is a focus of future work.

GCC2 is involved with linking LGs to the Golgi after NK cell activation, presumably without affecting the association of LGs with microtubules. This is in part supported by our experiments with Brefeldin-induced Golgi dispersion. In the absence of intact Golgi, LG convergence was more efficient. We propose that this improved convergence may be due to the absence of a physical barrier caused by the Golgi, allowing LGs to more tightly pack into the MTOC. That said, when the activating stimulus was removed from the cells with fragmented Golgi, the LGs were easily dispersed, while in the cells with intact Golgi, LGs remain converged (for at least 3 h after withdrawal of activating stimuli). This further emphasizes a role for the Golgi as an anchor for converged LGs that were approximated to the Golgi via dynein-mediated activation-induced LG convergence.

The utility of converged LGs has been shown in reductionist single- and multi-cell systems to promote killing efficiency and prevent non-directional degranulation and bystander cell killing. Using a recently developed three-dimensional complex cell arrangement system, TheCOS, we have extended this into simulated multicellular environments.^20^ Here, we found that, in a target/bystander or a more complex resistant tumor cell three-dimensional system, NK cells with converged LGs are focused upon killing their triggering target cell. When LG convergence was reduced via GCC2 absence, the dispersion of the LGs was maintained even when in the midst of complex cell arrangements. Furthermore, these cells with dispersed LGs were able to kill the triggering target cell, but also uncovered bystander killing of otherwise resistant tumor cells. Thus, the efficiency of convergence and non-directionality of LG dispersion could be seen in complex multilayer cell environments, with efficient convergence associated with more efficient target killing, and the lack of convergence (dispersion) making the NK cells prone to degranulate outside of the lytic synapse increasing bystander killing. Although rapid and excessive synaptic degranulation could theoretically result in bystander killing, as could the release of predocked LGs, the bystander killing of cells that do not promote NK cell activation and are resistant, such as osteosarcoma cells used in the present study (Figure S2A), suggests a direct role of LG dispersion (as prevented by GCC2) in bystander killing. This is reinforced by the observation that when Golgi are fragmented by Brefeldin A, convergence is more efficient and there is no bystander killing (Figure S1E) as well as the previously demonstrated concept that the contents of a single granule can be enough to induce target cell death.^3^

The impact of LG convergence and dispersion in complex three-dimensional environments questions the *in vivo* relevance of these cellular processes. Specifically, does the decreased on-target killing efficiency associated with dispersion create any host defense deficit? Having collected an 18-year cohort of individuals with NKD where a static deficit in NK cell activity results in clinically relevant immunodeficiency, this was a relevant place to search. This cohort has previously given rise to monogenic explanations for NKD and is characterized by susceptibility to viral infections, most notably those caused by herpesviruses and wart-causing viruses,^39–43^ and it has been complemented by NKD-related discoveries from other programs.^44–47^ Approximately one-quarter of our NKD cohort has been attached to a genetic explanation and many remain unsolved. Having identified two unrelated individuals in the NKD cohort with biallelic GCC2 variants led us to explore potential causality, but it certainly raised the possibility of the relevance of GCC2 and LG positioning in NK cell-mediated human host defense.

The two patients with biallelic GCC2 shared a common mutation, E1608G, which is located close to the GRIP domain. Hypothetically, this proximity could destabilize the CC-D structure (similar to predictions for the other private variants) and potentially alter interaction with key downstream effectors. When modeled in GCC2-deficient YTS cells, GCC2 E1608G failed to restore GCC2 function in enabling LG convergence. This was similar to what was found in *ex vivo* patient NK cells that demonstrated reduced killing efficiency and a failure to converge LGs in lytic synapses. The only other listed association for GCC2 is for a homozygous missense variant, R1669H, present in a patient with a severe neurological disease, although it was not clear if there was any immunological phenotype in that individual.^48^ Irrespective, a partial loss of GCC2 function is associated with a defect of LG movement and clinical presentation with NKD, suggesting an *in vivo* human relevance to killing efficiency and LG convergence.

The Golgi as a cellular center for production and recycling is well established but takes a new form and function via a role for the *trans*-Golgin GCC2. Specifically, it serves as “docking station” for diffusely localized cellular organelles that need to be compartmentalized in order to enable a specialized cell function. That said, it is unlikely that NK cell LGs represent GCC2’s only cargo, and exploration of similar paradigms for other lysosomal-related organelles where strict control of positioning and release is relevant remains to be investigated. Similarly, using the Golgi as a docking station for other diffusely localized cellular organelles after cell activation is potentially an appealing means to preserve cellular energy as opposed to relying upon constant motor protein activity to dictate organelle positioning. A role for the Golgi as a docking station for lysosome-related organelles in NK cells to facilitate their cytolytic function, however, governs the persistence of convergence after microtubule-directed LG delivery. In this context and through extension to a new human genetic disease, Golgi-GCC2 linkages demonstrate a mechanism for preventing bystander killing while promoting killing efficiency that is required for human host defense. Conversely, perhaps hardwired mechanisms to promote bystander killing by blocking convergence without interfering with degranulation could prove to be useful in improving the therapeutic use of cytotoxic cells against otherwise difficult-to-treat solid tumors where the killing of neighboring cells would be of great value.

### Limitations of the study

The major limitation of the study is that we have not established the molecular partner of GCC2 that enables its linkage to the LG governing its tethering function. Also, although we were able to link biallelic GCC2 variants to human NKD in two unrelated patients, additional cases would be instructive. Finally, although our work identifies a role for GCC2 in NK cell function, we do not know its specificity to NK cells or if it affects other cells that converge lysosome-related organelles such as cytotoxic CD8 T cells, mast cells, or melanocytes.

## RESOURCE AVAILABILITY

### Lead contact

Further information and requests for resources and reagents should be directed to and will be fulfilled by the lead contact, Jordan S. Orange (jso2121@cumc.columbia.edu).

### Materials availability

Materials are available from the lead contact upon reasonable request.

### Data and code availability

- Data reported are available from the lead contact upon request. Exome data have been deposited in AnVIL under the GREGoR Consortium dbGaP study ID phs003047. They are available upon request if access is granted. To request access, submit a data access request to dbGaP or through the Data Use Oversight System. In addition, summary statistics describing these data can be viewed on the GREGoR Consortium website (https://gregorconsortium.org/members/research-home/gregor-consortium-data) and the GREGoR Variant Browser (https://variants.gregorconsortium.org/) and are publicly available as of the date of publication. Relevant study design and enrollment information can be found at https://www.ncbi.nlm.nih.gov/projects/gap/cgi-bin/study.cgi?study_id=phs003047.v1.p1.
- This paper does not report original code.
- Additional information required to reanalyze the data reported is available from the lead contact upon request.

## STAR★METHODS

### EXPERIMENTAL MODEL AND STUDY PARTICIPANT DETAILS

#### Human PBMC

Human PBMC were obtained from whole blood from research volunteers after informed consent and enrollment in a research protocol approved by the institutional review board for the protection of human subjects of either Columbia University Irving Medical Center (AAAR7377) or Baylor College of Medicine (H-30487) and isolated by density centrifugation using Ficoll-Paque medium (Cytiva) as previously described.^41^ *ex vivo* NK cells were isolated from whole blood using RosetteSep Human NK Cell Enrichment Cocktail (Stemcell) according to manufacturer’s recommendations. For expansion and where indicated, primary NK cells were cultured in NK MACS Medium (Miltenyi) supplemented with 5% human AB serum (GeminiBio) and 500U/mL of IL-2 (Proleukin) for 2 weeks or until confluence.

#### Human subjects and exome sequencing

Individuals suspected of having NKD were enrolled in research protocols approved by the institutional review board for the protection of human subjects Columbia University Irving Medical Center (AAAR7377) and Baylor College of Medicine (H-30487) and clinical data evaluated and blood samples obtained for research NK cell studies and exome sequencing. Sequencing was performed and variants analyzed as described at Baylor College of Medicine.^49^

#### Cells and culture

The human NK cell line YTS and the MHC class-I-deficient 721.221 EBV-transformed B cell line were as originally described^50^ and maintained in RPMI 1640 (Thermo) complete media (R10) supplemented with 10% heat inactivated fetal bovine serum (Thermo), 10mM HEPES, 1mM sodium pyruvate, 2mM L-glutamine,1% non-essential amino acid, and 10U/mL penicillin/streptomycin (all from Thermo). The K562 erythroleukemia cell line was obtained from ATCC, and the LM7 and 143B osteosarcoma cells were the kind gift of Dr. Stephen Gottschalk (St. Jude, Memphis, TN) with the permission of Dr. Eugenie Kleinerman (MD Anderson, Houston, TX). Lenti-X 293T cells were obtained from Takara and cultured in DMEM Complete media (Thermo) (D10) supplemented with 10% heat inactivated fetal bovine serum, 10mM HEPES, 1mM sodium pyruvate, 2mM L-glutamine,1% non-essential amino acid, and 10U/mL penicillin/streptomycin. Cultured cells were maintained at 37°C and 5% CO2 at an average concentration of 5×10^5^ cells/mL and adherent cells or confluence of 80%.

### METHOD DETAILS

#### Gene editing and transduction

Crispr/CAS9 gene editing was performed using 2×10^6^ cells that were nucleofected with 5μM of sgRNA guide specific for GCC2 (Horizon; for the sequence, see Key resources table) and 5μg of Cas9-GFP mRNA (Horizon) in 100 μL of nucleofection Kit-R supplemented (Lonza) solution using the R-024 program of an Amaxa Nucleofector II (Lonza). After 24h, cells were sorted for GFP positivity, and single clones generated in 96-well plates were then selected based upon GCC2 expression evaluated by Western Blot.

Gene transduction was performed using lentiviral particles produced in HEK293T LENTI-X cells according to manufacturer’s instructions (Takara) using FuGene transfection reagent (Promega) in serum free DMEM (Thermo). Supernatants were collected and concentrated using PEG-it Virus Precipitation Solution (System Bioscience) and were used to transduce YTS cells via spinoculation. Lentiviral particles contained pCDH-CMV-MCS-EF1a-puro plasmids with GCC2 NM_181453.4 mRNA WT (kind gift from Dr. Suzanne Pfeffer from the Department of Biochemistry, Stanford University School of Medicine), the E1608G variant, or truncated GCC2 (amino acids 1–1532) fused with the GGGGSGGGGSGGGGS-GKKKKKKSKTKCVIM peptide. YTS cells were also transduced with a GALT-mCherry fusion protein containing the amino acids 1–81 from β1,4-galactosyltransferase (pmCherry-N1-GalT was a gift from Lei Lu, Addgene plasmid # 87327)^12^ or a fusion protein of pHluorin-LAMP1-mApple as previously reported.^3^ All constructs were generated or subcloned by Epoch Life Science custom cloning services (Sugarland, TX). After 72h, transduced cells were selected using progressively increasing concentrations of puromycin ending with 5 μg/ml.

#### Flow cytometric analyses

Cells were stained for flow cytometric analyses as described,^19^ and ~1×10^6^ cells were analyzed using a NovoCyte Penteon flow cytometer (at the Columbia Stem Cell Initiative Flow Cytometry Core Facility); this data were analyzed using FlowJo software. Flow cytometric data were plotted and statistically analyzed using Prism10 (GraphPad). Degranulation assay was performed with 5×10^4^ YTS and 721.221 target cells mixed for 90 min at 37°C after which cells were fixed and stained as described previously, using anti-CD56 to identify NK cells and anti-CD107a as a marker for degranulation.^19^ Detailed NK cell development was assessed using cryopreserved PBMC stained with surface antibodies as specified (antibodies utilized are listed in the Key resources table), followed by permeabilization using FoxP3 fixative buffer (Thermo) for 30 min on ice. Cells were then washed and incubated with antibodies diluted in FoxP3 permeabilization buffer for intracellular staining as specified (antibodies utilized are listed in the Key resources table).

#### Cytotoxicity assay

4h ^51^Cr-release assay using either K562 or 721.221 target cells was performed as described,^19^ using 1×10^4^ target cells per well of a 96-well U bottom plate. Effector cells were used in serial dilution starting at an effector to target cell ratio of 50:1 for PBMC or 10:1 for YTS cells. The activity of PBMC was measured using unstimulated cells or with 1000U/mL of IL-2 (Proleukin) added during the assay. After incubation, supernatants were transferred into Lumaplates (Revvity) and measured using a TopCountXL (PerkinElmer). Specific lysis (%) was calculated as: (experimental cpm – spontaneously released cpm)/(total cpm – spontaneously released cpm) ×100.

#### Confocal microscopy

For single cell or single cell conjugate confocal microscopy, 18-well chamber slides (Cellvis) were precoated with 5 mg/mL of mouse anti-human CD18 (IB4) (to adhere NK cells), a mix of mouse anti-human CD18 (IB4) and CD28 clone CD28.2 (Biolegend) (to adhere and activate NK cells), or mouse anti-human CD19 clone HIB19 (Biolegend) (to attach 721.221 targets). Either 2×10^4^ cells YTS cells or 4×10^4^ equally mixed YTS and 721.221 cells in 100μL of R10 media were added to each well and incubated for either 1h or 30 min at 37°C, respectively. The mixed YTS and 721.221 cells were preincubated in suspension for 30 min at 37°C prior to being added to the slide. After incubation, media was aspirated and cells were fixed with 4% paraformaldehyde in PBS, washed, and permeabilized with 0.25% of Triton X-100. Cells were then stained with fluorophore conjugated antibodies and Phalloidin (antibodies utilized are listed in Supplementary Table 2). For live cell imaging, YTS cells were labeled with Lysotracker Deep Red (Thermo) and 721:221 target cells with Cell Tracker Blue. 721.221 cells were added to chamber slides first and allowed to adhere for 30min, after which YTS cells were then added and images recorded every 5s to capture 100 frames (~8min). For all experiments, cells were visualized and images were acquired on a Zeiss Axioplan Observer Z1 with Yokigawa CSU-W1 T2 50μm spinning disk and 63X 1.49 NA objective. Excitation was by 405, 488, 505, 561, and 637 nm lasers and light captured via a Prime 95B metal oxide camera. Data were acquired with Slidebook software (Intelligent Imaging Systems) and analyzed using Imaris (Oxford instruments) or ImageJ (Fiji) software. LG distance to MTOC and LG distance to Golgi were measured as described for the mean granule distance method,^51^ with some modifications. In brief, 3D reconstructions were generated using Imaris software, and the tool for spots measurement was utilized to identify LGs and render the signal from Perforin and MTOC (using maximum α-Tubulin signal which has been validated as alternative to pericentrin^51^). For the Golgi rendering the tool for surface reconstruction was applied to the signal from mCherry-Galt. Upon 3D model reconstruction, the software was used to generate the shortest distance between the centroid of each spot (in the case of LG to MTOC distance) or the shortest distance between the LGs centroid and the surface of Golgi. The average of those distances were computed using at least 20 cells per measurement.

#### Lytic granule isolation and analysis

LGs were isolated from YTS cells using a Lysosome Enrichment Kit for Tissues and Cultured Cells (Thermo) according to manufacturer’s recommendations. In brief, 3×10^8^ cells were washed and sonicated, and mitochondria/cell debris were removed via centrifugation at 10000 × g. Supernatant was precipitated at 20000 × g, washed twice, resuspended, and layered on a percoll density gradient followed by ultracentrifugation at 340000 × g. LGs were harvested from the top layer and washed. For Western blot analysis of LG contents, they were lysed with NP40 lysis buffer (10mM HEPES pH8, 10mM KCl, 1mM EDTA pH 8, 0.4% NP40, 1X Halt proteinase phosphatase), separated using gradient 4–12% gel, and transferred to a nitrocellulose membrane. Membranes were blocked with skim milk, incubated with primary antibodies overnight (antibodies utilized are listed in the Key resources table), secondary antibodies for 1h, and imaged using an Odyssey CLx System (Licor). For surface biotinylation, LGs were incubated with EZ-Link Sulfo-NHS-SS-Biotin (Thermo) in PBS pH 8.0 at RT for 30min following manufacturer’s instructions, lysed, precleared, and then precipitated using streptavidin-agarose beads (Millipore) as we had previously described.^13^ Mass spectrometry was performed at the protein core facility of the Children’s Hospital of Philadelphia as described, and comparison of surface biotinylated to non-biotinylated LGs was performed.^13^ Only proteins identified in the lysates from surface biotinylated LGs in both of two independently repeated experiments with ≥3 identified peptide sequences were considered present.

#### Thermal collapse of strata (TheCOS)

The overarching strategy of TheCOS was to be able to bring into approximation specified ratios of cells on demand without having them be in contact *a priori*, while at the same time enabling direct visualization via microscopy as well as cell isolation at the end of an experiment. Full details and protocols are reported separately^20^ and are presented in brief and as relevant to present results. YTS cells, triggering 721.221 target cells and non-triggering target cells (K562 erythroleukemia, or LM7 or 143B osteosarcoma cells) were resuspended in 1% PolyN-isopropylacrylamide (PNIPAM) (Millipore Sigma) in Culture media at 4°C to enable 1:10 effector to target cell ratios with specified percentages of the different target cells. The individual cell suspensions were added (below the phase transition temperature of PNIPAM at 32°C) over a premade layer of 1% agarose hydrogel in culture media (lower layer) that had been previously poured into in a 3D-printed micromold assembly and allowed to cool and solidify. After adding a cell suspension in PNIPAM a 3D-printed piston was carefully inserted into the mircomold to create a flattened layer. The piston was then removed, exposing the newly created layer. This process was repeated for each subsequent layer and finally, a 1% agarose hydrogel at 45°C was added to fill the micromold and seal the cell strata. The entire sealed assembly was then placed on an isothermal plate (37–40°C) for 5 min to allow for the gelation of the cell suspension and collapse of the PNIPAM layers brining the layered cells into contact with each other within the agarose encasement. To assess the bystander killing capacity of NK cells, a three-layer cell strata of triggering target cell/YTS cell/non-triggering target cell was assembled. Following the collapse of the PNIPAM hydrogel, the aggregated cells encased in agarose were incubated for 4 h. Subsequently, the assembly was cut open through an incision guide printed into the micromold, and either imaged by placing the exposed matrix onto a coverslip or individual cells isolated by soaking the agarose matrix on PBS and filtering through a 40-μm cell strainer, after which flow cytometric analysis was performed using fluorescently-conjugated antibodies and live/dead staining reagents to measure the viability of specific cells.

### QUANTIFICATION AND STATISTICAL ANALYSIS

Numerical data were analyzed and displayed using GraphPad Prism 10.0 (GraphPad Software). D’Agostino and Pearson tests were used to assess normality, two-tailed student’s T test for significance with normal distributed data, and Mann-Whitney U tests for significance with non-normal data. Statistical significance of the difference between arbitrary curves such as those generated on ^51^Cr or in TheCOS were assessed using a modified Chi-squared method as previously reported.^52^ Values considered to be statistically significant were noted as follows: *p ≤ 0.05, **p ≤ 0.01, ***p ≤ 0.001, ****p ≤ 0.0001 and non-significant diferences are not shown.

## Supplementary Material

1

2

## Figures and Tables

**KEY RESOURCES TABLE:** 

REAGENT or RESOURCE	SOURCE	IDENTIFIER

Antibodies		

Alexa Fluor 647 anti-human Perforin	Biolegend	Cat# 308109, RRID:AB_493255
Alexa Fluor 488 anti-Tubulin-α Antibody	Biolegend	Cat# 627905, RRID:AB_893643
Anti-GCC2	Sigma-Aldrich	Cat# HPA035849, RRID:AB_10671941
GCC1 Polyclonal Antibody	Thermo Fisher Scientific	Cat# PA5-53988, RRID:AB_2641906
GOLGA4 Polyclonal Antibody	Thermo Fisher Scientific	Cat# PA5-76521, RRID:AB_2720248
GOLGA1 Polyclonal Antibody	Thermo Fisher Scientific	Cat# PA5-117970, RRID:AB_2902576
anti-human CD107a	Biolegend	Cat# 328602, RRID:AB_1134259
PE anti-human CD107a	Biolegend	Cat# 328608, RRID:AB_1186040
Anti-human CD18	Biolegend	Cat# 373402, RRID:AB_2650942
Anti-human CD28	Biolegend	Cat# 302943, RRID:AB_2616667
Anti-human CD19	Biolegend	Cat# 302202, RRID:AB_314232
Anti-Tubulin-α Antibody	Biolegend	Cat# 627902, RRID:AB_439761
Anti-Rab 6A	Santa Cruz	Cat# sc-81913, RRID:AB_1128894
Brilliant Violet 605 anti-human CD56 (NCAM)	Biolegend	Cat# 318333, RRID:AB_11142683
APC/Cyanine7 anti-human CD45	Biolegend	Cat# 368516, RRID:AB_2566376
Brilliant Violet 421 anti-human CD19	Biolegend	Cat# 302234, RRID:AB_11142678
Anti-β-Actin Antibody	Sigma-Aldrich	Cat# A5441, RRID:AB_476744
Brilliant Violet 711 anti-human CD3	Biolegend	Cat# 344838, RRID:AB_2565827
BUV395 CD94	BD Biosciences	Cat# 743954, RRID:AB_2741876
BUV496 CD3	BD Biosciences	Cat# 612940, RRID:AB_2870222
BUV496 CD14	BD Biosciences	Cat# 750381, RRID:AB_2874552
BUV496 CD19	BD Biosciences	Cat# 612938, RRID:AB_2870221
BUV563 CD56	BD Biosciences	Cat# 612928, RRID:AB_2870213
BUV805 CD45	BD Biosciences	Cat# 612891, RRID:AB_2870179
BV510 CD294	Biolegend	Cat# 350120, RRID:AB_2566446
BV650 NKp44	BD Biosciences	Cat# 744302, RRID:AB_2742132
BV711 CD117	Biolegend	Cat# 313230, RRID:AB_2566217
BV785 CD103	Biolegend	Cat# 350230, RRID:AB_2734364
FITC CD57	Biolegend	Cat# 359604, RRID:AB_2562387
PE Ki-67	Biolegend	Cat# 350504, RRID:AB_10660752
PE PLZF	BD Biosciences	Cat# 564850, RRID:AB_2738984
PE/Dazzle 594 CD34	Biolegend	Cat# 343534, RRID:AB_2564012
PE-Cy5 CD127	Biolegend	Cat# 351324, RRID:AB_10915554
PE-Cy7 T-bet	Biolegend	Cat# 644824, RRID:AB_2561761
PE-Cy7 CD158 (KIR2DL1/S1/S3/S5)	Biolegend	Cat# 339512, RRID:AB_2565579
PE-Cy7 CD158b/j (KIR2DL2/L3/S2)	Biolegend	Cat# 312609, RRID:AB_2563374
PECy-7 CD158e1 (KIR3DL1, NKB1)	Biolegend	Cat# 312720, RRID:AB_2563364
eFluor 660 EOMES	Thermo Fisher Scientific	Cat# 50-4877-42, RRID:AB_2574229
APC NKp80	Miltenyi	Cat# 130-112-591, RRID:AB_2653024
Alexa Fluor 700 CD16	Biolegend	Cat# 302026, RRID:AB_2278418
APC-Cy7 Zombie NIR	Biolegend	Cat# 423106

Biological samples		

Human PBMC from healthy donors	–	N/A
Human PBMC from NKD patients	–	N/A

Chemicals, peptides, and recombinant proteins		

CellTracker Blue CMAC Dye	Thermo Fisher Scientific	Cat# C2110
LysoTracker Deep Red	Thermo Fisher Scientific	Cat# L12492
Alexa Fluor^™^ Plus 405 Phalloidin	Thermo Fisher Scientific	Cat# A30104
MitoSpy^™^ Red CMXRos	Biolegend	Cat# 424801
Chromium-51 Radionuclide	revvity	Cat# NEZ030005MC
Brefeldin A Solution (1,000X)	Biolegend	Cat# 420601
Recombinant IL2	Clinigen	Proleukin
Poly(N-isopropylacrylamide)	Sigma-Aldrich	Cat# 535311-10G
UltraPure Agarose	Thermo Fisher Scientific	Cat# 16500500
RPMI 1640 Medium	Thermo Fisher Scientific	Cat# 11875093
DMEM, high glucose	Thermo Fisher Scientific	Cat# 11965092
HEPES (1M)	Thermo Fisher Scientific	Cat# 15630130
Sodium Pyruvate (100 mM)	Thermo Fisher Scientific	Cat# 11360070
L-Glutamine (200 mM)	Thermo Fisher Scientific	Cat# A2916801
MEM Non-Essential Amino Acids Solution (100X)	Thermo Fisher Scientific	Cat# 11140035
Penicillin-Streptomycin (5,000 U/mL)	Thermo Fisher Scientific	Cat# 15070063
Fetal Bovine Serum	Thermo Fisher Scientific	Cat# A5256801
Ficoll-Paque PLUS	Cytiva	Cat# 17144002
RosetteSep^™^ Human NK Cell Enrichment Cocktail	Stemcell	Cat# 15025
NK MACS^®^ Medium, human	Miltenyi	Cat# 130-114-429
Human Serum AB	GeminiBio	Cat# 100-612
Cas9 nuclease mRNA	Horizon	Cat# CAS12217
SgRNA GCC2 (CCAGATTGAAGCATCAGCTA)	Horizon	Cat# SG-013457-01-0002
SgRNA Rab6A (TCTAGTTCCACAATGTCCAC)	Horizon	Cat# SG-008975-01-0002
FuGENE^®^ HD	Promega	Cat# E2311
Puromycin Dihydrochloride	Thermo Fisher Scientific	Cat# A1113803
PEG-it Virus Precipitation Solution	System Biosciences	Cat# LV810A-1
eBioscience^™^ Foxp3/Transcription Factor Staining Buffer Set	Thermo Fisher Scientific	Cat# 00-5523-00
Chameleon^®^ Duo Pre-stained Protein Ladder	Licor	Cat# 928-60000
IRDye^®^ 800CW Goat anti-Mouse IgG Secondary Antibody	Licor	Cat# 926-32210
IRDye^®^ 680RD Goat anti-Rabbit IgG Secondary Antibody	Licor	Cat# 926-68071
IRDye^®^ 680RD Goat anti-Rat IgG Secondary Antibody	Licor	Cat# 926-68076
Poly(N-isopropylacrylamide)	Millipore sigma	Cat# 535311

Critical commercial assays		

Lysosome Enrichment Kit for Tissues and Cultured Cells	Thermo Fisher Scientific	Cat# 89839
Cell Line Nucleofector^™^ Kit R	Lonza	Ca# VCA-1001
PureLink^™^ HiPure Plasmid Maxiprep Kit	Thermo Fisher Scientific	Cat# K210006
TransDux^™^ MAX Lentivirus Transduction Reagent	SBI	Cat# LV860A-1
pPACKH1 HIV Lentivector Packaging Kit	SBI	Cat# LV500A-1
LumaPlate-96	Revvity	Cat# 6006633
18 Well chambered cover Glass	cellvis	Cat# C18-1.5H
Bolt^™^ Bis-Tris Plus Mini Protein Gels, 4–12%, 1.0 mm	Thermo Fisher Scientific	Cat# NW04120BOX
20X Bolt^™^ MOPS SDS Running Buffer	Thermo Fisher Scientific	Cat# B0001
Bolt^™^ Transfer Buffer (20X)	Thermo Fisher Scientific	Cat# BT0006
EZ-Link Sulfo-NHS-SS-Biotin	Thermo Fisher Scientific	Cat#21331
Streptavidin–Agarose from Streptomyces avidinii	Millipore Sigma	Cat# S1638-1ML

Deposited data		

Exome sequence	dbGaP/NHGRI GREGoR Consortium	phs003047.v1.p1
Experimental models: Cell lines		
YTS Parental	–	N/A
YTS GCC2 KO	Generated in house	N/A
YTS GCC2 KO + GCC2 WT	Generated in house	N/A
YTS GCC2 KO + GCC2 K777I	Generated in house	N/A
YTS GCC2 KO + GCC2 Q851P	Generated in house	N/A
YTS GCC2 KO + GCC2 E1608G	Generated in house	N/A
YTS Parental + GCC2-KRAS	Generated in house	N/A
YTS GCC2 KO + GCC2-KRAS	Generated in house	N/A
YTS Parental + GalT-mCherry	Generated in house	N/A
YTS GCC2 KO + GalT-mCherry	Generated in house	N/A
YTS Parental + pHluorin-LAMPI-mApple	Generated in house	N/A
YTS GCC2 KO + pHluorin-LAMPI-mApple	Generated in house	N/A

Recombinant DNA		

pCDH-CMV-EF1a-puro (backbone)	System Biosciences	Cat# CD510B-1
pCDH-CMV-GCC2WT-EF1a-puro	Epoch Life Science	Designed in House
pCDH-CMV-GCC2K777I-EF1a-puro	Epoch Life Science	Designed in House
pCDH-CMV-GCC2Q851P-EF1a-puro	Epoch Life Science	Designed in House
pCDH-CMV-GCC2E1608G-EF1a-puro	Epoch Life Science	Designed in House
pCDH-CMV-GCC2-KRAS-EF1a-puro	Epoch Life Science	Designed in House
pCDH-CMV-GalT-mCherry-EF1a-puro	Epoch Life Science	Designed in House
pCDH-CMV-pHluorin-LAMP1-mApple-EF1a-puro	Epoch Life Science	Designed in House

Software and algorithms		

Imaris 10.1	Oxford Instruments	N/A
Fiji	Public domain	N/A
Prism 10.0	GraphPad Software	N/A
Flowjo 10.8	Becton Dickinson	N/A

## Highlights

- The Golgi serves as a regulator of lytic granule convergence in natural killer cells
- GCC2 is present on LGs and helps them tether to the Golgi to sustain convergence
- KO of GCC2 results in LG dispersion, non-directional degranulation, and bystander killing
- Humans with biallelic GCC2 missense variants have NK deficiency
