# Supplementary material for: The Golgi complex governs natural killer cell lytic granule positioning to promote directionality in cytotoxicity
Source: Cell Rep. Author manuscript; Available in PMC 2025 Feb 21. (PMC11844255; doi:10.1016/j.celrep.2024.115156)

Cell Reports, Volume *44*

Supplemental information

The Golgi complex governs natural killer cell lytic granule positioning to promote directionality in cytotoxicity

Luis A. Pedroza, Frederique van den Haak, Alexander Frumovitz, Evelyn Hernandez, Everardo Hegewisch-Solloa, Tabitha K. Orange, Keri B. Sheehan, Susan Prockop, Aaron Bodansky, Ivan K. Chinn, James R. Lupski, Jennifer E. Posey, Emily M. Mace, Yu Li, and Jordan S. Orange

Methods S1: Clinical histories and presentations for patients P1 and P2, related to Figure 6.

Patient 1

Patient 1 is a male second child of healthy parents, born after an uneventful pregnancy and without perinatal complications. He had rhinovirus at 2mos of age with 1mo of persistent symptoms, and respiratory syncytial virus at 5mos of age requiring hospitalization. At age 10 and 12mos he was hospitalized for respiratory compromise and presumed respiratory viral infection. Owing to the severity and recurrence of illness with fevers he had several immunological assessments performed that suggested accentuated/prolonged physiologic IgG nadir vs hypogammaglobulinemia. Evaluations for primary antibody deficiency were normal and his IgG levels ultimately normalized by 17 months with documentation of intact vaccine-specific IgG. Lymphocyte subset analyses were normal, but NK cell function was persistently low. Respiratory symptoms persisted over his first 3 years. He also had skin rashes starting at age 1 which ultimately resolved at age 4 when he was diagnosed with molluscum contagiosum. Initial research NK cell studies performed at age 1 documented low cytotoxicity without or with added IL-2 stimulation in vitro compared to the range of over 200 healthy donors (graphs) as well as NK cell percentages within normal ranges. This pattern continued through last evaluation at age 7.

**Age**	12 months	13 months	14 months
**Lymphocyte subsets**	#/%	#/%	#/%
CD3+	5116/68	3780/72	4090/70
CD4+	3160/42	2468/47	2601/45
CD8+	1808/24	1260/24	1412/24
CD19+	2257/30	1313/25	1578/27
CD16+/56+	1/75	1/53	2/92
CD4:CD8	1.7	1.9	1.9
**NK cell cytotoxicity**			
50:1	8 (L)	8 (L)	6 (L)
25:1	4 (L)	5 (L)	2 (L)

12.5:1	3 (L)	3 (L)	1 (L)
6:1	1	0 (L)	0 (L)
NK LU	0.9 (L)	1 (L)	0.3 (L)

#/%: absolute number and percentage of lymphocytes.
NK LU: Lytic units
(L): below the normal levels for age.

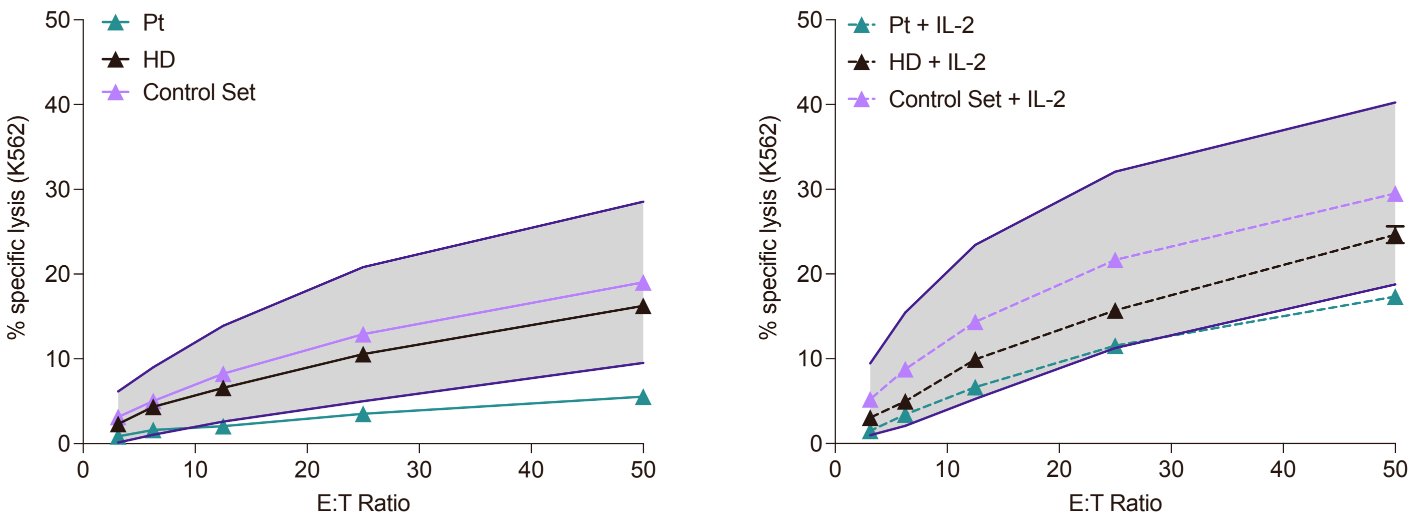

Patient 2

Patient 2 is a Male first child of healthy unrelated parents. He experienced early life upper respiratory infections and otitis media and had tonsillectomy/adenoidectomy at age 15 for recurrent pharyngitis. At age 16 he presented with prolonged fever, fatigue and meningismus and was diagnosed with Lyme meningitis, receiving 1mo of ceftriaxone treatment. Later that year he developed myalgias and diffuse arthralgias and was diagnosed with acute EBV infection followed by several months of persistent symptoms. A clinical immunological evaluation was performed and demonstrated normogammaglobulinemia and no protective titers against 23 pneumococcal polysaccharides. After receiving a 23-serotype pneumococcal polysaccharide vaccine, only 10 serotypes had titers ≥1.3 μg/ml and in light of his history he was started on

immunoglobulin replacement therapy. Lymphocyte subsets demonstrated a skewed CD4/CD8 T cell ratio, but otherwise normal cell counts, and NK cell cytotoxic function was low (see table). He was referred for research evaluation at age 19 and initial studies demonstrated low NK cell cytotoxicity without or with added IL-2 stimulation in vitro compared to the range of over 200 healthy donors (graphs), as well as NK cell percentages within normal ranges.

**Age**	17 years	18 years	21 years
**Lymphocyte subsets**	#/%	#/%	#/%
CD3+	1668/78	1466/74	1164/73
CD4+	605/36	475/32	493/31
CD8+	793/47	561/38	496/31
CD19+	313/14	276/14	181/11
CD16+/56+	125/5	232/11	234/15
CD4:CD8	0.44	0.85	1
**NK cell cytotoxicity**			
50:01:00	3 (L)		
25:01:00	1 (L)		
12.5:1	1 (L)		
6:01	0		
NK LU	0.9 (L)		

#/%: absolute number and percentage of lymphocytes.
NK LU: Lytic units
(L): below the normal levels for age.

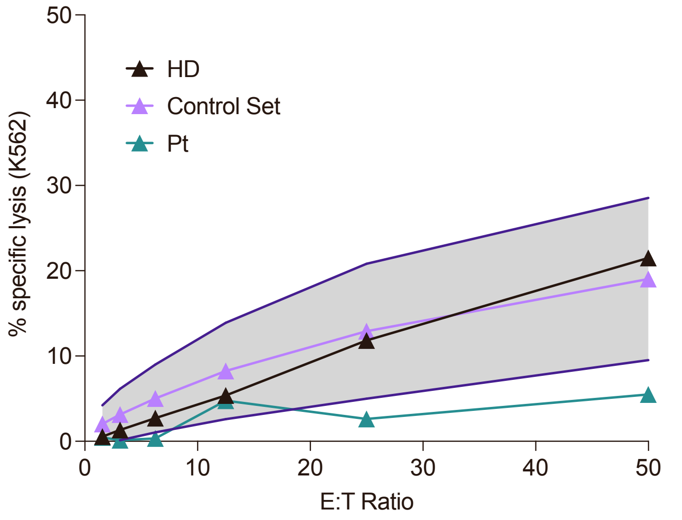

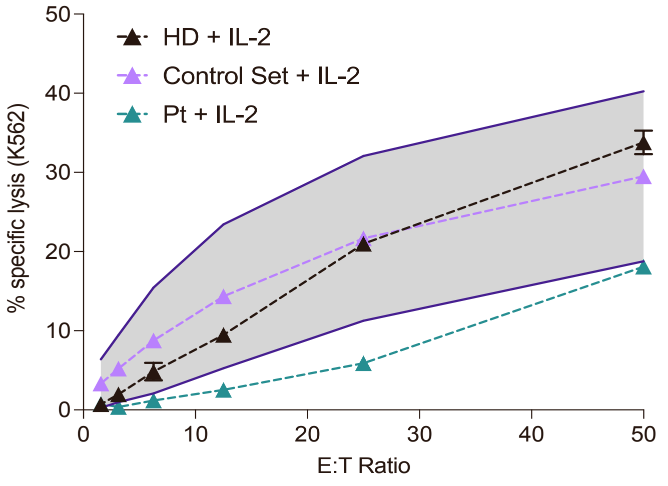

Supplementary Fig. S1. Control experiments for NK cell activation, LG isolation, bystander killing and degranulation.

(A) LG convergence to the MTOC is driven only by activating anti-CD18 monoclonal antibodies. LG convergence to MTOC mediated by different clones of anti-CD18 or anti-CD28 fixed to glass surfaces. Glass bottom 18-well plates were coated with different monoclonal antibodies, and parental YTS cells were added and incubated at 37°C for 1h. Cells were then fixed, permeabilized, and stained for F-actin (phalloidin-Alexa Fluor 405), LGs (anti-perforin-Alexa Fluor 488), and the MTOC (α-tubulin-Alexa Fluor 592). Mean distance of LGs to the MTOC was measured from 20 to 25 cells per condition obtained from 3 independent experiments. The non-activating anti-CD18 mAb clone IB4 was used as basis for cell attachment without LG convergence (non-activation control). Anti-CD28 was used as a positive activation control for LG convergence. Noted comparisons were statistically different (*=p<0.05, Mann-Whitney U test). **(B) Quality control for Golgi contamination during LG isolation.** Protein extracts from isolated LGs using the same procedure as that utilized for LG proteomic analysis were blotted for Giantin (a cis-Golgi protein) (top) and CD107A as a marker for LGs (bottom). **(C) "Bystander cells" are resistant to direct killing by YTS cells.** Representative 4h ^{51}Cr release killing assay of parental YTS cells against susceptible 721.221 target (green), and resistant target cells K562, 143B and SaoS-LM7. **(D) Degranulation assay using LAMP1-Phluorin reporter.** YTS parental cells or YTS GCC2 KO cells transduced an mApple-LAMP1-pHluorin reporter were stimulated on a glass surface coated with anti-CD18(IB4) and anti-CD28 for 1 hour and imaged every 2 minutes. The number of pHluorin transitions on the glass surface were manually counted and plotted for YTS parental (red) and YTS GCC2 KO (blue). Noted comparisons were statistically different using a non-parametric T test with Welsh correction (*=p<0.05 Mann-Whitney U tests). **(E) Bystander Killing by Brefeldin A treated cells.** YTS

cells were pretreated with 1μM of Brefeldin for 1 hour and then TheCOS stacks were generated, incubated and digested. Isolated cells were stained for flow cytometric analysis using LIVE/DEAD Near IR and directly conjugated antibodies anti-CD56**, -**CD45, and -CD19 to allow for identification of live and dead effector, target, and bystander cells using the gating strategy utilized in Fig. 3A. Representative results of resistant K562 target cell “bystander” killing in TheCOS via flow cytometry by untreated YTS (green) or Brefeldin-A treated YTS cells (blue) with increasing percentages of susceptible 721.221 cells (0 to 80%) of the target cells in the TheCOS stack.

Supplementary Fig. S2. Specificity of Golgi and LG interaction.

(A) GCC2 requirement for LG proximity to Golgi fragments. YTS Parental or YTS GCC2 KO cells stably expressing GALT-mCherry, were pre-incubated for 1 hour with 1ug/ml of Brefeldin A, washed twice and stimulated on a glass surface coated with anti-CD18(IB4) and anti-CD28 for 1 hour. Cells were fixed and stained with Phalloidin (orange), and anti-Perforin (yellow),and then imaged using spinning disk confocal microscopy and Golgi visualized via mCherry (teal). Untreated cells (left) compared to those that were Brefeldin A-treated allow for the visualization of Golgi fragmentation. Magnified insets from the Brefeldin A-treated cells show approximation of Golgi fragments to LGs primarily when GCC2 was present (top) and not in GCC2 KO cells (bottom) (scale bar = 10μm). **(B) quantitative analysis LG and Golgi fragment approximation with or without GCC2.** Brefeldin A-treated YTS parental or GCC2 KO cells shown in the images were analyzed and the LG distance to Golgi fragments were quantified using Imaris Software. At least 40 cells per condition for YTS parental (yellow) and GCC2 KO (green) were measured and the mean distance in each cell plotted as an individual point. Noted comparisons were statistically different (*=$p<0.05$ Mann-Whitney U tests). **(C) Golgi-dependent convergence is specific to LGs.** YTS Parental cells were stained with Mitospy (teal),

pretreated with Brefeldin A and stimulated on a glass surface coated with anti-CD18(IB4) and anti-CD28 for 1 hour. Cells were fixed, permeabilized and stained with Phalloidin (orange) and anti-Perforin (yellow) and imaged using confocal microscopy. In each case, activation induced the convergence of LGs while the positioning of mitochondria was diffuse and unaffected by NK cell activation or Brefeldin A treatment, (scale bar = 10µm). **(D) Quantitative analysis for the specificity of LG convergence and Brefeldin A effect.** Cells from the images and at least 20 others were evaluated for the distance of the LGs to the MTOC (left) and Mitochondria distance to the MTOC (right) using Imaris software. The mean distance in individual cells of each was plotted in both untreated and Brefeldin A-treated cells in the presence of only non-activating adhesion anti-CD18(IB4) or anti-CD28-induced activation. Noted comparisons were significant (****= $p<0.0001$,**= $p<0.01$, *= $p<0.05$), or not significant (ns) as determined using Mann-Whitney U tests.

Supplementary Fig. S3. *In-silico* analysis of patient-derived mutations.

(A) *In-silico* protein modeling of patient mutations. GCC2 protein sequence (Uniprot Q8IWJ2) was used to generate predicted homodimer structures using Alphafold2, which were visualized using Molstar. Each residue (WT left column and mutants right column) was modeled within 50 amino acids in each direction with enlargements to show details of relevant residues. **(B) Protein alignment of mutated residues.** GCC2 protein sequence within 10 amino acids of each of the mutated residues were aligned against the predicted sequence from pig, mouse, Chicken and frog using Clustal default coloring. **(C) In silico scoring of deleteriousness for patient-derived mutations.** In silico prediction of the deleteriousness of each of the patient-derived mutations was obtained from CADD and polyPhen-2, and the evolutionary conservation was retrieved from PhyloP.

Supplementary Fig. S4. Endogenous expression of patient-derived mutations and reconstitution analyses in GCC2 KO YTS cells. (A) GCC2 expression in patient and healthy donor (HD) ex vivo cells. Whole cell lysate of PBMC from patients 1 and 2 as well as 5 different HD were evaluated for GCC2 (top) and GAPDH (bottom) protein levels by Western blot analysis. The relative expression of GCC2 in each sample (corrected for GAPDH loading) relative to HD2 was measured and is shown. **(B) GCC2 expression in patient cells.** Purified ex vivo NK cells from a healthy control or patient 2 were fixed, permeabilized and staining with fluorophore-conjugated Phalloidin (red) and anti-GCC2 (detected using secondary anti rabbit-AF647, teal). Cells were then imaged using confocal microscopy, (scale bar = 10µm). (**C) Killing efficiency of YTS GCC2 KO cells reconstituted with WT GCC2 or patient-derived GCC2 variants.** Representative 4h ^{51}Cr release killing assay against susceptible 721.221 target using either parental YTS cells or YTS cells stably reconstituted with full length GCC2 WT or each of the patient derived GCC2 variants. Noted comparisons were significantly different (****=p<0.0001,***=p<0.001, **=p<0.01 or *=p<0.05 Chi-squared tests). **(D) Presence of variant GCC2 protein on LGs in reconstituted YTS cells.** Whole cell lysates and purified LGs (left and right in each panel, respectively) from parental YTS cells (P) or GCC2 KO YTS cells expressing either the GCC2-KRAS, GCC2-WT, GCC2 K77I, GCC2 Q815P, or GCC2 E1608G constructs. Evaluation of GCC2 (top) and CD107a (bottom) levels by Western blot analysis were performed and in each the enrichment of CD107a signifies the effective purification of the LGs relative to the whole cell lysate.

Supplementary Fig. S5. Extended NK cell phenotyping in GCC2 NKD patients.

Flow cytometric analysis of PBMC from GCC2 NKD patients and a healthy donor evaluating NK cell developmental subsets. (A) Uniform manifold approximation and projection (UMAP) analysis of NK cell developmental subsets (Lineage: $CD3^{-}CD14^{-}CD19^{-}$) with expression data overlaid from 1 healthy donor and patients 1 and 2. (B) Frequency of each NK cell developmental subset (Stages 4-6) gated on $CD45^{+}Lin^{-}LiveCD94^{+}$ with bars showing the mean of 3 distinct healthy donors ±SD and 2 GCC2 patients ±range with points showing the individual values (red=healthy donors, black=GCC2 patients). Frequency of NK cell developmental subsets positive for (C) KIRs [CD158a/b/e1/g/h/j], (D) Granzyme B, (E) TBET, and (F) EOMES. Bars display the mean of 3 distinct healthy donors ±SD and 2 GCC2 patients ±range with points showing the individual values (red=healthy donors, black=GCC2 patients).

Supplementary Fig. S6. Effect of Rab6A deletion in YTS cells.

(A) Validation of Rab6A deletion in YTS cells KO cells. Whole cell lysate from YTS parental or Rab6A CRISPR CAS9 KO cells were evaluated using anti-Rab6A antibody (top) and anti-GAPDH (bottom) via Western blot analysis. **(B) Localization of LGs and Golgi in Rab6A KO cells**. YTS parental or Rab6A KO cells were attached on anti-CD18(IB4), or stimulated using anti-CD18(IB4)/anti-CD28-coated glass surfaces for 40 minutes and then fixed, permeabilized and stained using Phalloidin, anti-Perforin, anti-α-Tubulin and anti-Giantin. Representative images using confocal microscopy and showing the Golgi (teal), LGs (yellow) and filamentous actin (red) are shown after activation in a parental (left) and Rab6A KO cell, (scale bar = 10µm). **(C) LG distance to MTOC in Rab6A KO cells.** Cells from all of the conditions in B (adhered or activated, in parental or Rab6A KO cells) were evaluated for the positioning of the LGs relative to the MTOC after activation using Imaris software. Noted comparisons were either significant (****=$p<0.0001$) or not significant, (ns) via Mann-Whitney U tests.

Supplementary Fig. S1

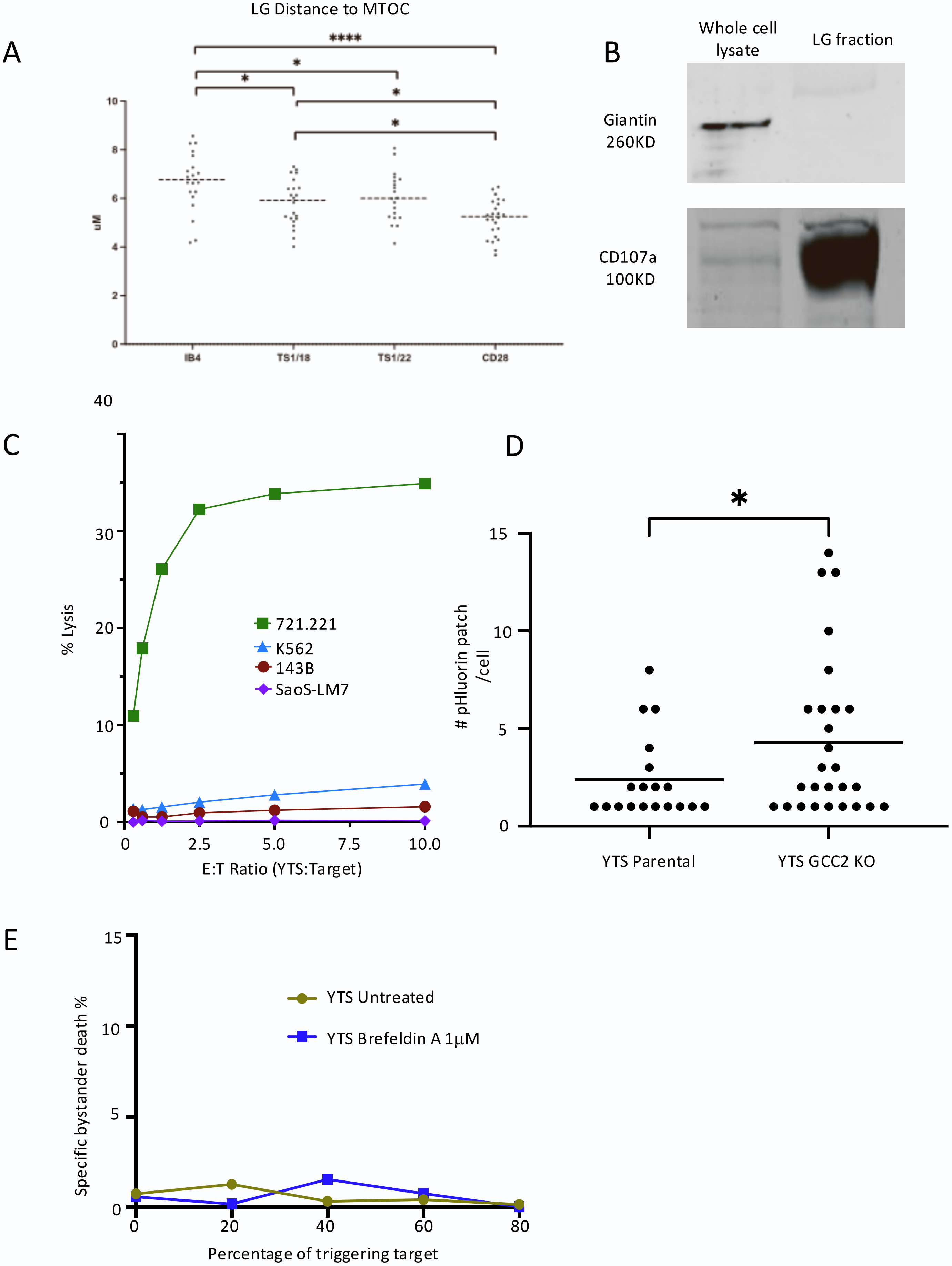

Supplementary Fig. S2

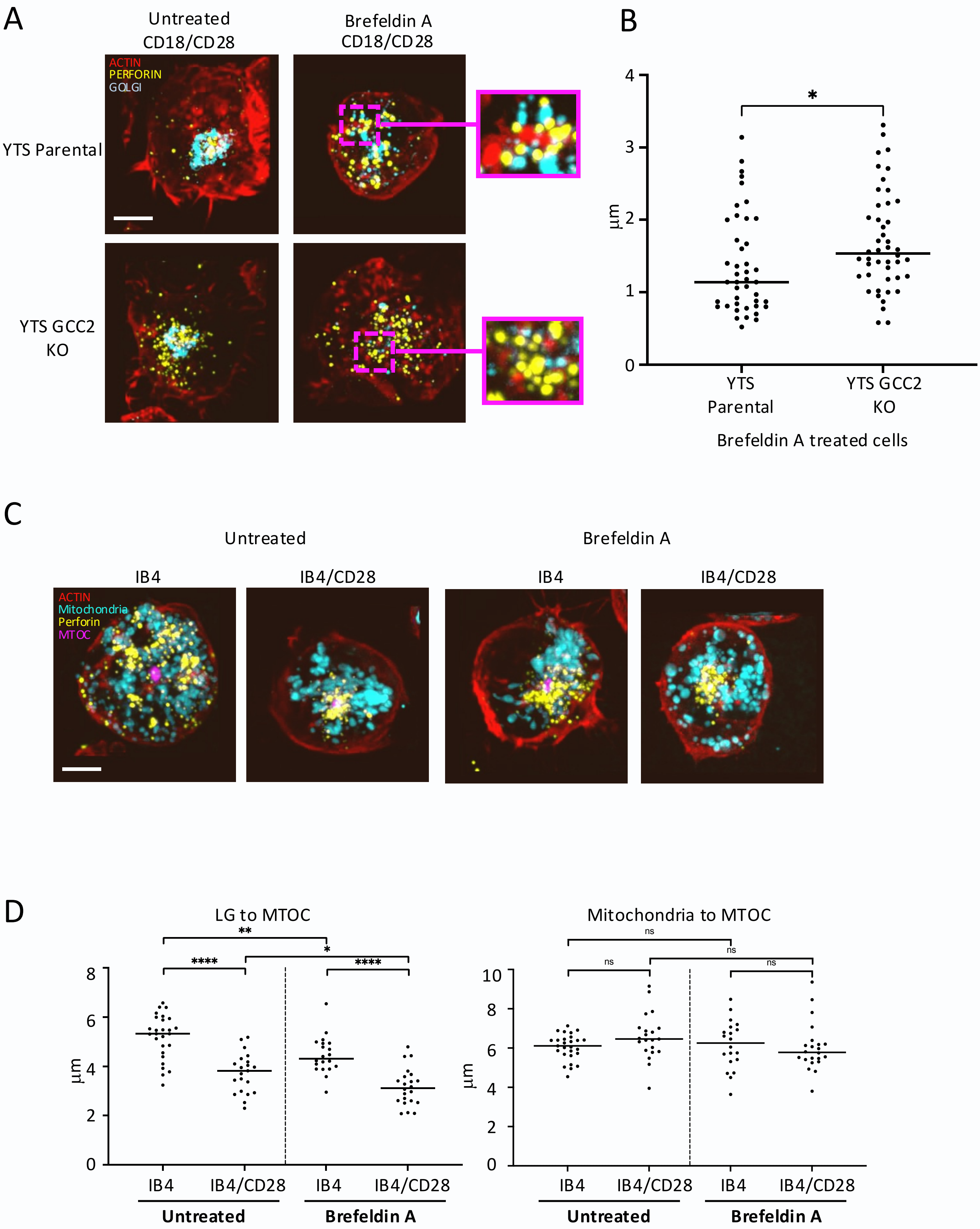

Supplementary Fig. S3

A

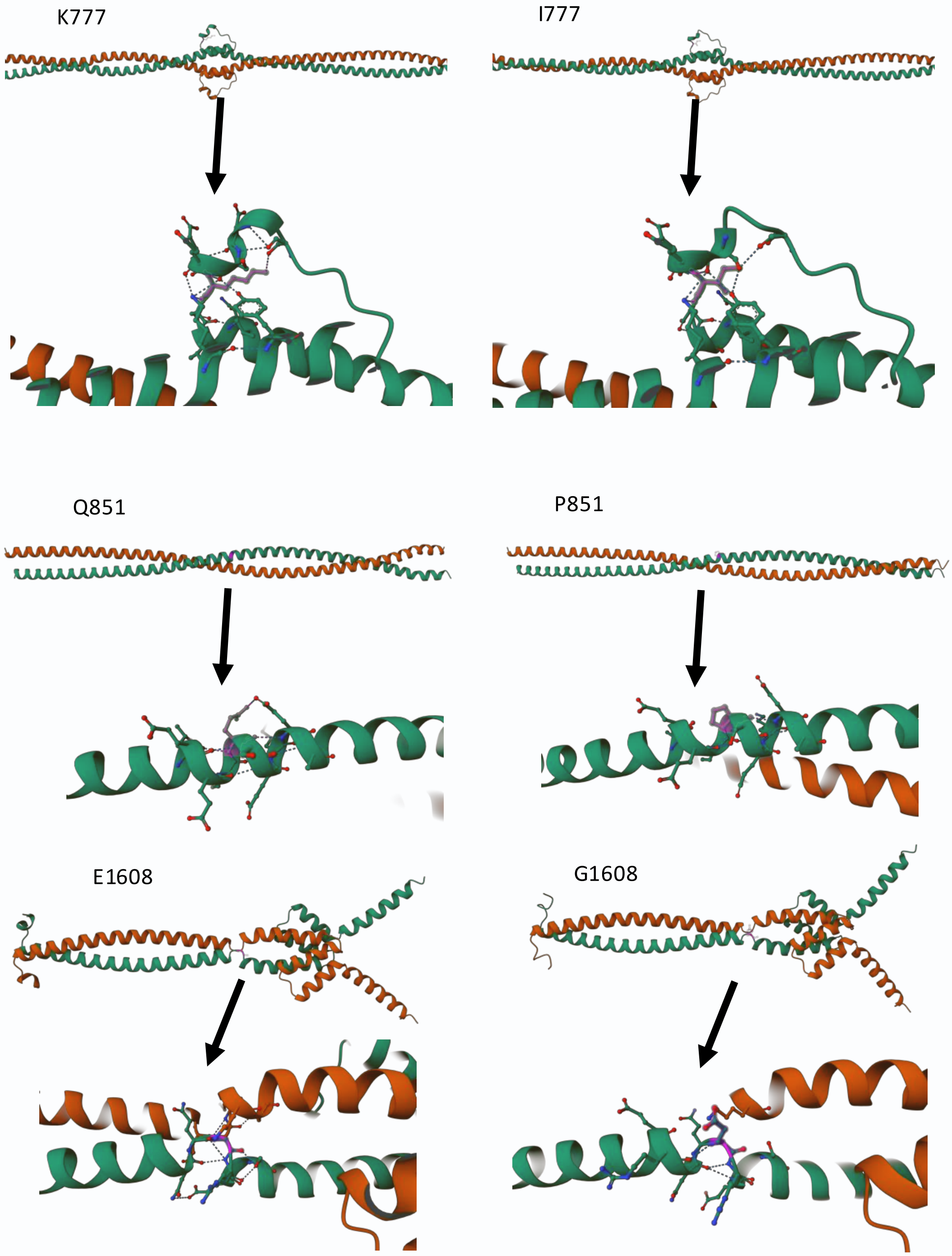

B

```
           767                   787  841                  861  1598                 1618
Human      GSEVSEDSEEKDVVNVLQAVG      VLLRKELEEIQSEKEALQSDL      SEIRRLERNQEREKSAANLEY
Pig        ESKVSEESEDQDIVNVLQAVS      VTLRKELEETLSKKEALEHDL      SEIRRLERNQEREKSAANLEY
Mouse      RASILEENEEEDVVKLIQAVG      ILLRKELDAVTSAKEALQLDL      SEIRRLERNQEREKSVANLEY
Chicken    GYSISDSNEECDFLNLLQIAN      DHLKEELEEVLSEKEALQHDI      SEIRRLERNQEREKSVANLEY
Frog       RETLSIDDAEQDIIHMLQSIN      VLLKEQLDETIRDKETLLNDL      NEVRRLERNQEREKSVANLEY
              :  .. : *.::::* :.        *:::*:     **:*  *:    .*:************.*****
```

C

	CADD	PhyloP	Polyphen2
**K777I**	19.5	2.84	0.229
**Q851P**	18.4	0.527	0.122
**E1608G**	28.1	3.67	1

Supplementary Fig. S4

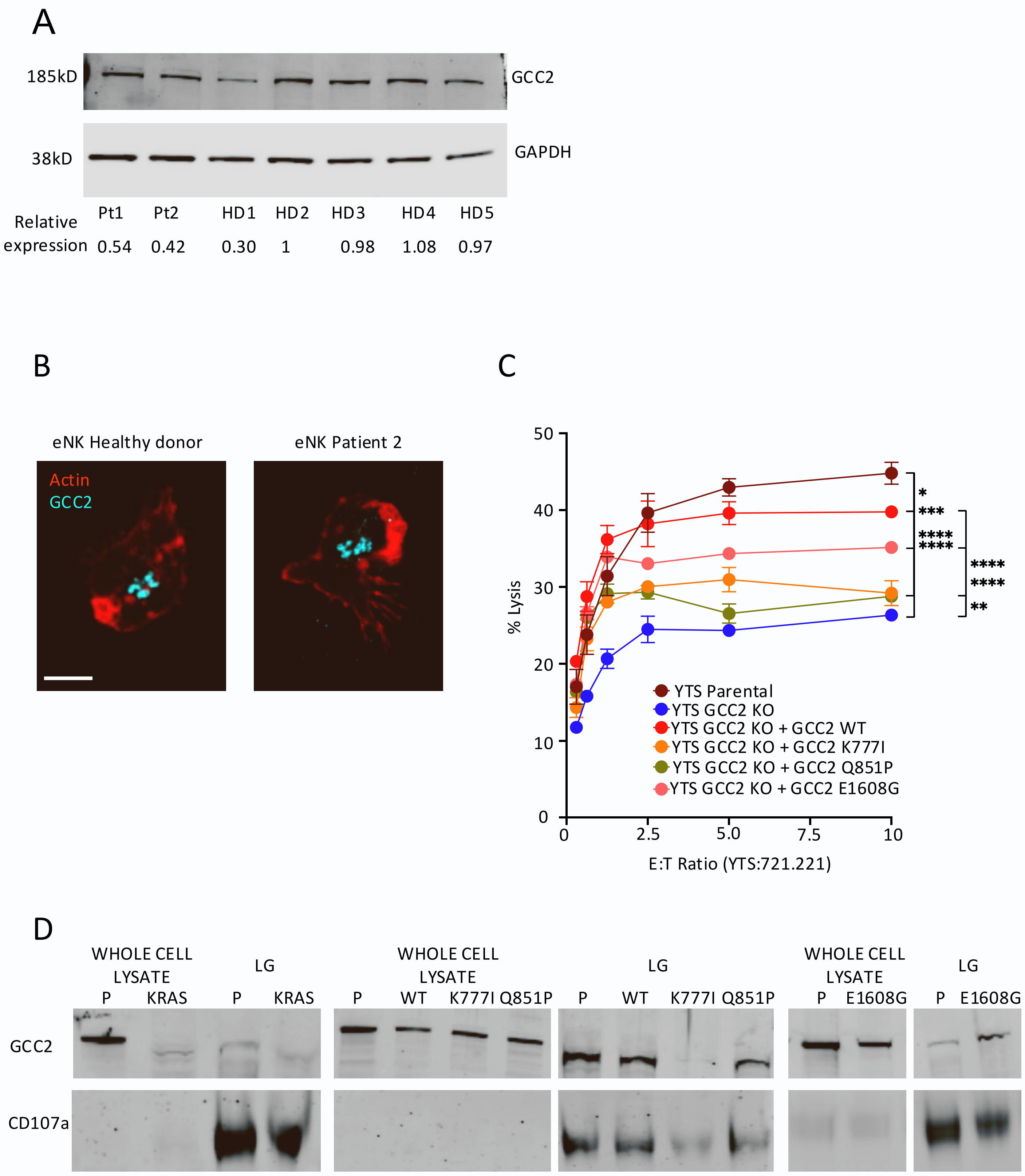

Supplementary Figure S5

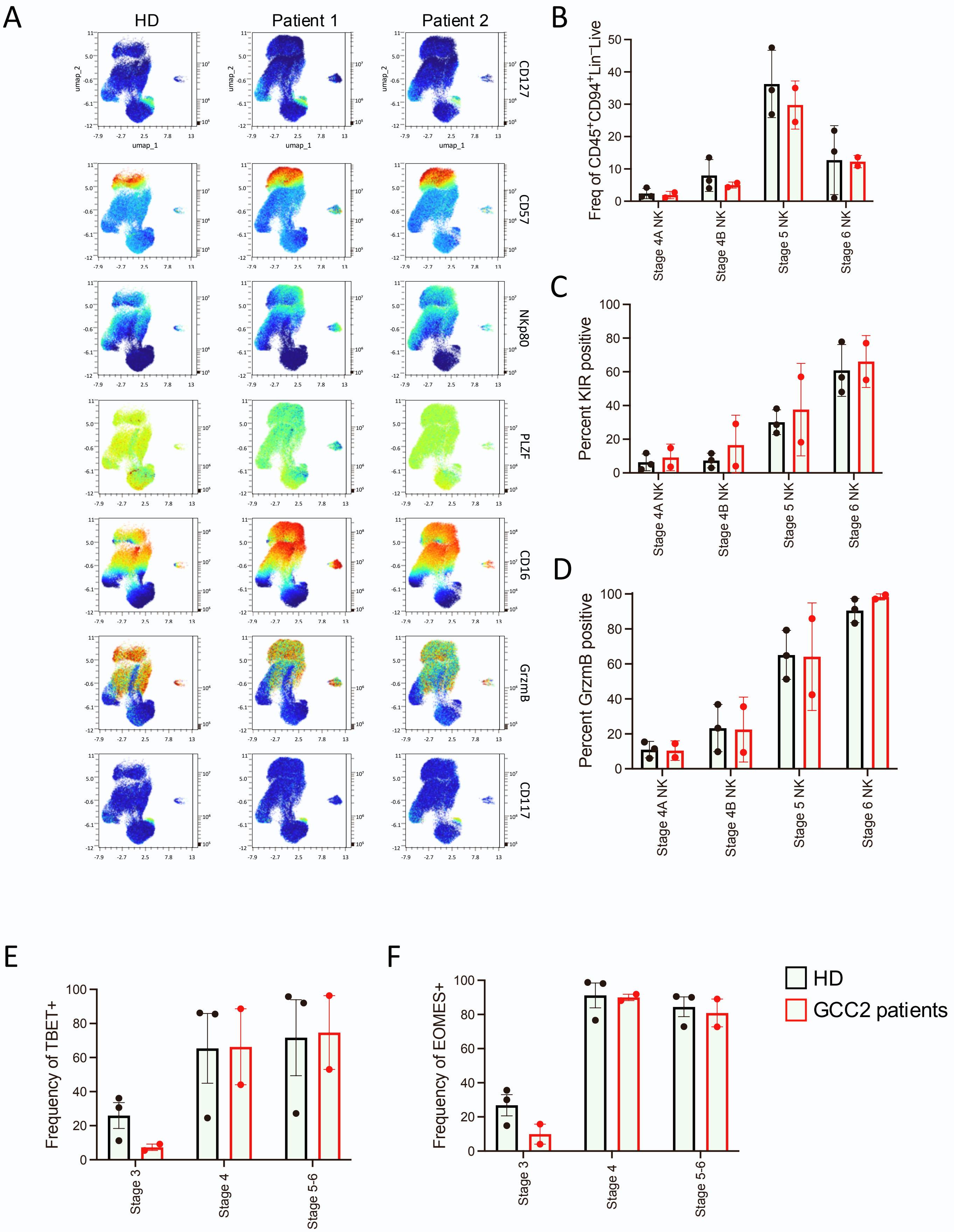

Supplementary Fig. S6

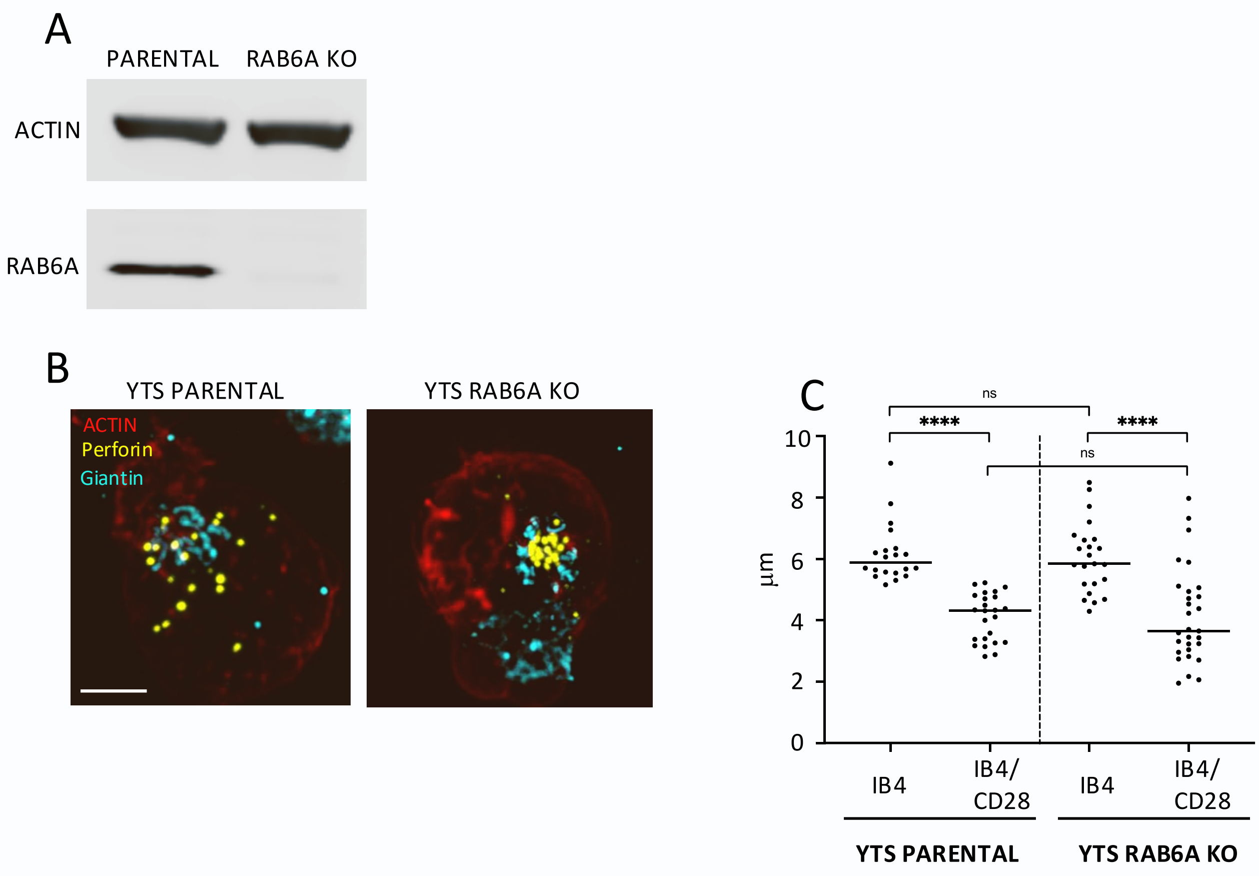

Supplement: 1 [file NIHMS2052752-supplement-1.pdf]
